# Global burden, regional disparities, and future projections of hypertensive kidney disease in older adults: analysis of GBD 1990–2021 data

**DOI:** 10.3389/fneph.2025.1656865

**Published:** 2025-10-17

**Authors:** Juan Li, Zeyu Jiao, Fang Cheng, Ting Liu, Ruixia Kang, Yongyuan Cai, Ruifang Zhang, Xiaoming Xue

**Affiliations:** 1 Department of Cardiology, Shanxi Traditional Chinese Medical Hospital, Taiyuan, Shanxi, China; 2 The Institute of Plant Protection, Chinese Academy of Agricultural Sciences, Beijing, China; 3 Department of Bioinformatics, Beijing Pineal Diagnostics Co., Ltd., Beijing, China; 4 Guang’anmen Hospital, China Academy of Chinese Medical Sciences, Beijing, China

**Keywords:** hypertensive kidney disease, Global Burden of Disease (GBD), health inequalities, Joinpoint regression, risk factor, prediction

## Abstract

**Aims:**

Hypertensive kidney disease (HKD) contributes significantly to global morbidity and mortality. This study evaluated the burden of HKD in older adults (≥60 years) across 204 countries from 1990 to 2021 and projected trends to 2045.

**Methods:**

Data from the Global Burden of Disease Study 2021 were used to estimate HKD prevalence, incidence, mortality, and disability-adjusted life years (DALYs). Age-standardized rates (ASRs) were calculated with 95% uncertainty intervals (UIs). Temporal trends were analyzed using Joinpoint regression. Slope and concentration indices quantified health inequality. Decomposition and frontier analyses explored burden drivers. Future projections were made using Nordpred-based Bayesian age-period-cohort models. Sensitivity analyses assessed model robustness. Risk-attributable mortality was also estimated.

**Results:**

In 2021, global ASRs were 1674.9 (prevalence), 93.4 (incidence), 36.5 (mortality), and 631.1 (DALYs) per 100,000 older adults. High-SDI regions had higher prevalence (ASPR: 1857.8) and incidence (ASIR: 126.5), while low-SDI regions showed higher mortality (ASMR: 58.6) and DALY rates (ASDR: 972.7). Males across all age groups had higher prevalence (e.g. 95 plus: 9109.6 vs. 7031.5 per 100,000). Leading risk factors included low fruit intake (6.98 deaths per 100,000), high sodium, and lead exposure. From 1990-2021, ASIR (AAPC = 0.63%), ASMR (0.99%), and ASDR (0.77%) rose, while ASPR declined (-0.25%). Decomposition attributed burden increases mainly to population growth (72.3%) and aging (6.7%). Frontier analysis revealed substantial room for improvement in middle-SDI countries. Sensitivity analyses confirmed the stability of trend estimates and projections. Forecasts indicate that deaths in adults ≥90 will triple by 2045 (e.g. 95 plus: 75,271 vs. 20,242 in 2021).

**Conclusion:**

HKD burden has grown substantially, with persistent geographic and socioeconomic disparities. Effective mitigation requires not only demographic- and region-specific interventions but also improved access to early detection and dietary risk reduction. Integration of kidney care into primary health systems and aging-focused strategies will be crucial to curb future disease escalation.

## Graphical Abstract

**Figure:**
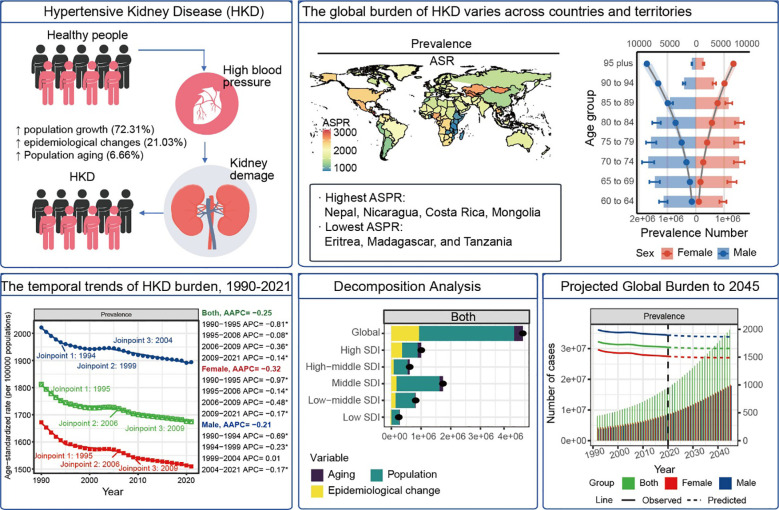

## Introduction

Hypertensive Kidney Disease (HKD) is a major cause of chronic kidney disease and significantly contributes to increased cardiovascular risk (1, 2). In the United States, recent data from the US Renal Data System indicate that hypertension is responsible for over 30% of end-stage renal disease (ESRD) cases, underscoring its significant clinical and economic burden (3). In China, the kidney disease data system indicates that HKD ranks as the third leading cause of ESRD, following primary glomerular diseases and diabetic nephropathy. Consequently, HKD represents a significant public health challenge (4–6).

The Sustainable Development Goals (SDGs) and the WHO Global Action Plan have set a target of reducing premature mortality from noncommunicable diseases (NCDs) by one-third by 2030. However, a recent global analysis revealed that by 2021, only 23% of countries had fully implemented core NCD control strategies, with many nations significantly lagging behind their targets (7). HKD, as a crucial factor in both kidney and cardiovascular disease progression, presents a substantial challenge to achieving this goal, as it accelerates mortality and contributes to long-term adverse health outcomes. Addressing HKD through targeted interventions is essential to alleviating the burden of both kidney and cardiovascular diseases (8).

Evaluating the epidemiological landscape of HKD is vital for effective health system planning (9). However, global epidemiological evaluations of HKD have been limited historically. This study aims to analyze current trends in the burden of HKD worldwide, examine how population and epidemiological factors have influenced changes in the disease burden over the past 32 years, and predict future trends in HKD burden by 2045.

## Methods

### Ethics statement

This study is based on publicly available data and does not involve human or animal subjects; therefore, ethical approval is not required.

### Data sources and modeling framework

Data on HKD burden were obtained from the Global Health Data Exchange (GHDx) GBD Results Tool (http://ghdx.healthdata.org/gbd-results-tool). Metrics analyzed included prevalence, incidence, mortality, and disability-adjusted life years (DALYs), along with 95% uncertainty intervals, stratified by age, sex, year, and region. The GBD 2021 methodology has been detailed in prior publications (10). Briefly, input data sources were identified and harmonized through systematic data extraction and curation. In regions with sparse or missing data, estimates were smoothed using spatiotemporal Gaussian process regression (ST-GPR). Bias due to variation in study methods and case definitions was corrected using meta-regression with Bayesian regularization and trimming (MR-BRT). Core epidemiological parameters of HKD - such as incidence, prevalence, remission, and cause-specific mortality - were modeled using DisMod-MR 2.1, a Bayesian meta-regression framework. Models assumed negative binomial likelihoods to account for overdispersion and were estimated via Markov Chain Monte Carlo (MCMC), with standard convergence diagnostics applied to evaluate model fit.

### Statistics

#### Definitions and SDI stratification

Older adults were defined as individuals aged ≥60 years, and age was stratified into five-year intervals. Countries were assigned to 21 distinct GBD regions based on geographic and epidemiological similarities (11). The Socio-demographic Index (SDI), an aggregate measure of development, was constructed from metrics including income per capita, educational attainment, and fertility rates (12). SDI scores range between 0 and 1, with higher values indicating more advanced development. Based on these scores, regions were divided into five categories: low (<0.45), low-middle (0.45–0.60), middle (0.60–0.69), high-middle (0.69–0.80), and high (≥0.80) (13). Trends over time were evaluated using the Average Annual Percentage Change (AAPC) along with corresponding 95% confidence intervals (14).

### Joinpoint regression

Joinpoint regression is a statistical method employed to detect significant changes in temporal trends by segmenting data into continuous linear pieces connected at inflection points, known as joinpoints. This technique facilitates the identification of periods where the rate of change in a variable significantly shifts (15). In this analysis, we employed version 5.2.0 of the Joinpoint Regression Program (developed by the National Cancer Institute, Rockville, MD, USA) to examine global trends in age-standardized prevalence (ASPR), incidence (ASIR), mortality (ASMR), and disability-adjusted life year (ASDR) rates related to HKD from 1990 to 2021. Statistical significance of the detected joinpoints was assessed using the Monte Carlo permutation test, with a threshold of p < 0.001 (16). The maximum number of joinpoints was set to 3. Outputs included Annual Percentage Change (APC) for each segment and Average Annual Percentage Change (AAPC) for overall trends. Positive values of APC or AAPC indicate an increasing trend, while negative values signify a decreasing trend (17). To ensure robustness, sensitivity analyses were conducted by varying the number of joinpoints (from 3 to 4) in Joinpoint regression. Results were compared in terms of AAPC direction, statistical significance, and model fit (AIC).

### Health inequalities

Health disparities refer to quantifiable differences in health outcomes observed across population groups stratified by social, economic, geographic, or demographic characteristics (18).To evaluate inequality in the burden of HKD, we applied two metrics: the Slope Index of Inequality (SII) and the Concentration Index. The Lorenz curve was constructed based on population-weighted cumulative SDI rankings. Where component data for SDI were incomplete, imputation strategies described above were applied (19, 20).

### Decomposition analysis

To quantify the drivers of change in DALYs between 1990 and 2021, we employed the Das Gupta decomposition method, partitioning the total change into contributions from population growth, population aging, and epidemiological shifts (21).

### Frontier analysis

To evaluate the efficiency of healthcare systems, we utilized frontier analysis by comparing the HKD burden to that of top-performing regions with similar levels of sociodemographic development. A non-parametric data envelopment method was employed to generate a non-linear efficiency frontier, indicating the lowest possible burden attainable given a region’s developmental context. This analysis incorporated ASDR and the SDI from 1990 to 2021, with robustness enhanced through 1,000 bootstrap replications (22).

### Projections using Nordpred

For future projections, we employed the Nordpred method, an age-period-cohort (APC) model, which combines the powerful capabilities of the generalized linear model (GLM) (23). To predict epidemiological indicators of HKD from 2021 to 2045. The model assumes stationary age and cohort effects while projecting linear trends from the last ten years of observed data. It can model data through the GLM structure to accurately estimate the impact of age, period, and cohort on epidemiological indicators (24). The number of joinpoints in the APC curves was limited to one to avoid overfitting (23, 24). Forecast validity was evaluated by training the model on 1990–2016 data and comparing predictions for 2017–2021 against observed GBD estimates.

All analyses were conducted using R version 4.3.3 and StataMP 16. A p-value of less than 0.05 was considered statistically significant.

## Results

### The overall burden of HKD in the elderly population

In 2021, the global prevalence of HKD was estimated at 17,536,443 cases (95% UI: 15,545,348 to 19,663,169), with an ASPR of 1,674.94 per 100,000 population (95% CI: 1,486.02 to 1,876.68). The global incidence of HKD reached 997,968.1 cases (95% UI: 748,213.5 to 1,279,361), corresponding to an ASIR of 93.41 per 100,000 (95% CI: 69.92 to 119.79). The number of deaths attributed to HKD was 370,912.9 (95% UI: 254,239.7 to 496,361.7), with an ASMR of 36.45 per 100,000 (95% CI: 25.02 to 48.72). The global DALYs burden for HKD was 6,639,950 cases (95% UI: 4,767,109 to 8,737,395), with an ASDR of 631.06 per 100,000 (95% CI: 453.45 to 829.01) (**Tables 1**, **2**, **Supplementary Table S1**).

**Table 1:** Age-standardize prevalent rates and incident rates for HKD in 2021 for both sexes and rate changes from 1990 to 2021.

Location	Prevalence_ASPR_1990	Prevalence_ASPR_2021	Prevalence_AAPC_1990_2021	Incidence_ASIR_1990	Incidence_ASIR_2021	Incidence_AAPC_1990_2021
Global	1812.28(1602.79 to 2049.42)	1674.94(1486.02 to 1876.68)	-0.25(-0.26 to -0.24)	76.92(54.51 to 102.3)	93.41(69.92 to 119.79)	0.63(0.62 to 0.63)
Age Group						
95 plus	7975.68(7199.93 to 8788.89)	7608(6855.96 to 8351.13)	-0.26(-0.28 to -0.24)	56.75(39.41 to 74.76)	44.67(33.14 to 57.04)	0.76(0.73 to 0.77)
60 to 64	703.17(613.7 to 815.11)	649.66(569.77 to 741.7)	-0.33(-0.36 to -0.31)	84(62.16 to 107.96)	69.07(51.73 to 90.56)	0.65(0.63 to 0.66)
65 to 69	1089.18(953.07 to 1245.9)	981.53(860.74 to 1112.53)	-0.3(-0.32 to -0.27)	112.5(80.01 to 149.86)	105.06(82.07 to 132.15)	0.71(0.68 to 0.73)
70 to 74	1712.82(1499.86 to 1957.98)	1555.37(1367.21 to 1756.15)	-0.21(-0.22 to -0.2)	141.71(100.66 to 190.18)	141.39(106.73 to 178)	0.76(0.73 to 0.79)
75 to 79	2529.28(2228.12 to 2845.02)	2354.17(2080.94 to 2622.83)	-0.22(-0.22 to -0.21)	152.29(106.83 to 209.65)	171.53(125.31 to 220.95)	0.6(0.58 to 0.62)
80 to 84	3564.97(3178.85 to 3974.28)	3328.14(2984.32 to 3686.17)	-0.21(-0.22 to -0.21)	147.35(104.08 to 198.55)	161.12(117.08 to 211.57)	0.16(0.14 to 0.19)
85 to 89	4817.18(4340.43 to 5380.53)	4505.31(4031.36 to 5015.82)	-0.19(-0.2 to -0.17)	168.01(98.25 to 251.99)	153.94(113.34 to 196.58)	0.14(0.12 to 0.16)
90 to 94	6266.91(5623.97 to 7014.55)	5891.56(5356.29 to 6538.22)	-0.15(-0.16 to -0.14)	35.14(24.39 to 47.07)	189.23(114.46 to 271.62)	0.35(0.29 to 0.38)
Sex						
Female	1672.6(1480.54 to 1889.26)	1509.92(1341.39 to 1690.21)	-0.32(-0.33 to -0.31)	70.25(49.61 to 93.58)	85.45(63.89 to 109.89)	0.64(0.63 to 0.64)
Male	2021.89(1784.54 to 2287.12)	1894.35(1676.38 to 2125.82)	-0.21(-0.22 to -0.2)	86.32(61.33 to 114.7)	103.83(77.81 to 133.08)	0.6(0.59 to 0.61)
Both	1812.28(1602.79 to 2049.42)	1674.94(1486.02 to 1876.68)	-0.25(-0.26 to -0.24)	76.92(54.51 to 102.3)	93.41(69.92 to 119.79)	0.63(0.62 to 0.63)
SDI Region						
High SDI	1875.27(1663.05 to 2115.61)	1857.79(1654.77 to 2076.5)	-0.04(-0.05 to -0.02)	112.98(81.14 to 149.37)	126.53(95.62 to 162.01)	0.37(0.34 to 0.39)
High-middle SDI	1664(1465.92 to 1883.86)	1428.43(1260.84 to 1603.94)	-0.49(-0.51 to -0.47)	63(44.44 to 83.83)	83.67(62.2 to 107.66)	0.93(0.91 to 0.94)
Middle SDI	1856.28(1624.37 to 2107.43)	1654.79(1452.76 to 1864.69)	-0.36(-0.39 to -0.33)	59.84(41.35 to 80.73)	84.81(62.99 to 108.94)	1.13(1.11 to 1.14)
Low-middle SDI	1822.93(1604.24 to 2070.07)	1725.98(1520.23 to 1943.38)	-0.2(-0.21 to -0.18)	49.67(33.88 to 67.37)	71.68(51.47 to 94.27)	1.17(1.14 to 1.2)
Low SDI	1666.37(1459.51 to 1885.41)	1587.87(1396.14 to 1790.32)	-0.17(-0.17 to -0.16)	42.56(29.39 to 57.15)	56.94(40.86 to 75.17)	0.95(0.93 to 0.97)
GBD Region						
High-income Asia Pacific	1918.71(1699.9 to 2174.19)	1840.35(1637.78 to 2069.26)	-0.14(-0.16 to -0.13)	117.71(84.61 to 155.48)	130.25(96.42 to 169.24)	0.4(0.34 to 0.44)
High-income North America	2127.41(1874.15 to 2424.26)	2254.84(1984.59 to 2552.58)	0.2(0.17 to 0.22)	131.25(91.09 to 177.2)	142.07(104.4 to 186.36)	0.27(0.24 to 0.31)
Western Europe	1706.85(1504.81 to 1931.1)	1613.13(1425.02 to 1809.96)	-0.18(-0.19 to -0.17)	109.02(77.66 to 144.56)	119.61(90.65 to 152.86)	0.33(0.32 to 0.35)
Australasia	1765.75(1521.96 to 2025.85)	1669.73(1428.84 to 1939.1)	-0.23(-0.3 to -0.19)	127.86(95.09 to 166.88)	147.09(107 to 190.5)	0.49(0.46 to 0.52)
Andean Latin America	1223.32(1048.62 to 1424.42)	1250.12(1073.38 to 1436.25)	0.09(0.07 to 0.1)	60.15(40.28 to 83.25)	114.79(81.75 to 153.45)	2.15(2.1 to 2.19)
Tropical Latin America	1922.85(1673.51 to 2196.67)	1924.35(1683.85 to 2190.89)	0.01(-0.01 to 0.03)	64.77(44.52 to 87.21)	93.95(68.75 to 122.36)	1.18(1.13 to 1.23)
Central Latin America	2326.62(2046.07 to 2635.44)	2330.05(2072.61 to 2594.05)	0.01(0 to 0.01)	84.08(56.78 to 114.92)	122.25(90.94 to 157.24)	1.23(1.22 to 1.25)
Southern Latin America	1220.2(1035.57 to 1415.34)	1222.4(1054.34 to 1410.41)	0(-0.03 to 0.02)	95.76(65.87 to 129.45)	130.61(94.75 to 172.15)	1.01(0.98 to 1.04)
Caribbean	1398.91(1222.9 to 1594.06)	1376.03(1205 to 1552.01)	-0.05(-0.06 to -0.04)	52.4(35.2 to 71.55)	87.55(62.26 to 116.02)	1.69(1.68 to 1.71)
Central Europe	1521.26(1343.86 to 1724.05)	1475.03(1314.27 to 1636.6)	-0.09(-0.1 to -0.09)	45.28(31.09 to 61.33)	85.97(62.65 to 111.97)	2.09(2.04 to 2.14)
Eastern Europe	1634.91(1431.9 to 1856.64)	1579.98(1385.01 to 1787.97)	-0.11(-0.11 to -0.1)	25.87(16.94 to 36.11)	46.98(32.55 to 63.08)	1.94(1.92 to 1.96)
Central Asia	2629.15(2319.74 to 2972.75)	2619.69(2295.95 to 2954.81)	-0.01(-0.03 to 0)	25.74(16.55 to 35.96)	49.82(34.27 to 67.66)	2.16(2.11 to 2.21)
North Africa and Middle East	1603.5(1416.46 to 1816.23)	1573.06(1391.41 to 1769.52)	-0.05(-0.06 to -0.04)	93.17(63.72 to 126.6)	153.08(110.01 to 201.96)	1.64(1.61 to 1.67)
South Asia	1857.46(1632.72 to 2112.43)	1679.13(1475.66 to 1901.79)	-0.36(-0.38 to -0.33)	45.73(30.94 to 62.32)	63.78(45.49 to 84.49)	1.07(1.05 to 1.1)
Southeast Asia	1799.17(1564.27 to 2056.5)	1857.23(1624.59 to 2111.52)	0.13(0.11 to 0.15)	50.67(34.44 to 69.2)	78.31(55.45 to 103.6)	1.44(1.41 to 1.47)
East Asia	1721.03(1498.58 to 1967.56)	1314.38(1142.23 to 1495.67)	-0.84(-0.88 to -0.79)	59.12(41.43 to 79.3)	71.09(53.88 to 90.7)	0.58(0.55 to 0.6)
Oceania	1618.31(1387.8 to 1866.5)	1605.95(1379.48 to 1847.51)	-0.02(-0.03 to -0.01)	39.42(26.25 to 54.44)	53.21(37.13 to 72.18)	0.98(0.97 to 1)
Western Sub-Saharan Africa	2118.01(1866.31 to 2384.93)	2040.32(1801.13 to 2289.48)	-0.13(-0.13 to -0.12)	49.23(34.38 to 65.92)	65.87(47.29 to 87.08)	0.94(0.92 to 0.96)
Eastern Sub-Saharan Africa	914.9(784.98 to 1054.6)	894.38(770.31 to 1026.87)	-0.08(-0.1 to -0.06)	36.41(25.13 to 49.15)	46.05(32.91 to 60.84)	0.77(0.75 to 0.79)
Central Sub-Saharan Africa	2347.2(2021.78 to 2692.84)	2300.29(2009.84 to 2600.8)	-0.06(-0.07 to -0.06)	34.66(23.36 to 47.64)	48.13(33.98 to 64.06)	1.07(1.06 to 1.08)
Southern Sub-Saharan Africa	2192.11(1914.88 to 2494.54)	2247.23(1955.92 to 2551.49)	0.09(0.08 to 0.11)	61.24(41.84 to 82.96)	85.42(61.16 to 111.37)	1.07(1.02 to 1.1)

AAPC, the Average Annual Percentage Change (AAPC); ASPR, age-standardized prevalence rates; ASIR, age-standardized incidence rates; ASMR, age-standardized mortality rates; ASDR, age-standardized disability-adjusted life year (DALYs) rates.

**Table 2:** Age-standardize death rates and DALYs for HKD in 2021 for both sexes and rate changes from 1990 to 2021.

Location	Death_ASDR_1990	Death_ASDR_2021	Death_AAPC_1990_2021	DALY_ASIR_1990	DALY_ASIR_2021	DALY_AAPC_1990_2021
Global	27.27(18.08 to 37.06)	36.45(25.02 to 48.72)	0.99(0.94 to 1.02)	501.18(353.92 to 664.43)	631.06(453.45 to 829.01)	0.77(0.73 to 0.8)
Age Group						
95 plus	8.27(5.42 to 11.77)	10.45(7.11 to 14.61)	0.77(0.75 to 0.8)	278.35(196.02 to 382.22)	344.31(244.76 to 464.13)	0.71(0.69 to 0.72)
60 to 64	12.26(8.03 to 17.2)	15.26(10.37 to 21.22)	0.7(0.63 to 0.78)	348.79(243.25 to 474.41)	424.07(303.25 to 579.77)	0.65(0.59 to 0.7)
65 to 69	20.71(12.86 to 28.85)	24.53(15.95 to 33.19)	0.6(0.54 to 0.66)	480.49(323.62 to 647.6)	559.05(393.22 to 741.74)	0.54(0.49 to 0.6)
70 to 74	32.44(20.24 to 43.8)	42.5(28.18 to 56.47)	0.89(0.82 to 0.95)	608.36(423.19 to 790.94)	774.2(550.83 to 994.58)	0.72(0.64 to 0.77)
75 to 79	53.14(36.79 to 72.26)	70.84(50.11 to 93.49)	0.95(0.86 to 1.03)	791.48(576.24 to 1029.11)	1014.17(749.32 to 1302.81)	0.81(0.75 to 0.87)
80 to 84	101.12(70.23 to 131.21)	139.34(100.63 to 177.07)	1.11(0.99 to 1.21)	1187.83(869.05 to 1493.97)	1557.26(1155.31 to 1953.36)	0.96(0.87 to 1.02)
85 to 89	160.61(110.89 to 212.59)	239.39(166.01 to 316.47)	1.4(1.33 to 1.46)	1668.64(1264.43 to 2148.33)	2327.1(1701.87 to 2983.37)	1.16(1.11 to 1.21)
90 to 94	224.43(146.4 to 309.82)	371.39(243.62 to 528.77)	1.66(1.52 to 1.79)	2150.22(1498.74 to 2890.21)	3300.64(2246.8 to 4519.6)	1.41(1.29 to 1.53)
Sex						
Female	21.88(14.35 to 30.19)	30.56(20.55 to 41.54)	1.13(1.08 to 1.17)	405.2(282.96 to 544.88)	528.99(373.97 to 699.25)	0.89(0.85 to 0.92)
Male	36.07(23.82 to 50.03)	44.95(31.18 to 59.98)	0.75(0.69 to 0.79)	642.4(451.11 to 871.01)	765.53(550.45 to 1003.76)	0.59(0.55 to 0.62)
Both	27.27(18.08 to 37.06)	36.45(25.02 to 48.72)	0.99(0.94 to 1.02)	501.18(353.92 to 664.43)	631.06(453.45 to 829.01)	0.77(0.73 to 0.8)
SDI Region						
High SDI	17.31(11.49 to 23.51)	29.06(21.09 to 37.84)	1.74(1.57 to 1.86)	308.68(223.48 to 402.33)	470.43(359.48 to 591.77)	1.4(1.27 to 1.5)
High-middle SDI	19.59(12.54 to 27.55)	23.74(15.42 to 33.24)	0.66(0.58 to 0.72)	364.03(255.36 to 488.38)	411.41(288.82 to 555.44)	0.43(0.37 to 0.47)
Middle SDI	41.8(28.04 to 56.69)	46.55(31.29 to 62.85)	0.37(0.34 to 0.41)	731.04(513.79 to 972.33)	809.63(570.51 to 1072.34)	0.33(0.3 to 0.36)
Low-middle SDI	34.41(22.17 to 49.47)	42.66(27.96 to 58.97)	0.73(0.68 to 0.79)	644.21(438.11 to 900.84)	773.76(532.06 to 1048.05)	0.66(0.62 to 0.69)
Low SDI	57.93(37.75 to 80.7)	58.58(38.57 to 80.55)	0.08(0.03 to 0.12)	1012.62(672.44 to 1396.41)	972.72(653.09 to 1333.43)	-0.1(-0.13 to -0.08)
GBD Region						
High-income Asia Pacific	14.57(9.49 to 21.01)	12.57(7.24 to 19.15)	-0.54(-0.64 to -0.41)	247.88(176.97 to 336.41)	214.5(142.18 to 305.07)	-0.51(-0.6 to -0.42)
High-income North America	24.83(17.1 to 32.31)	58.79(44.51 to 72.66)	2.96(2.75 to 3.14)	423.2(307.19 to 540.18)	893.19(701.04 to 1091.75)	2.54(2.28 to 2.83)
Western Europe	12.45(7.51 to 18.59)	17.68(11.21 to 25.53)	1.34(1.2 to 1.46)	231.65(159.4 to 319.56)	273.62(190.56 to 374.77)	0.68(0.6 to 0.79)
Australasia	13.58(9.93 to 17.92)	18.61(11.51 to 27.12)	1.06(0.8 to 1.26)	210.99(161.54 to 267.39)	261.6(171.98 to 368.77)	0.66(0.47 to 0.81)
Andean Latin America	64.87(41.57 to 92.27)	88.78(54.78 to 129.73)	1.22(1.12 to 1.33)	1056.17(681.79 to 1510.84)	1407.74(870.51 to 2060.23)	1.11(1.03 to 1.19)
Tropical Latin America	38.8(25.5 to 53.1)	43.86(28.16 to 60.56)	0.58(0.47 to 0.71)	692.49(478.18 to 932.59)	763.83(515.83 to 1038.14)	0.44(0.36 to 0.52)
Central Latin America	48.21(30.32 to 68.51)	65.19(40.02 to 94.11)	0.96(0.8 to 1.12)	810.81(540.11 to 1130.74)	1170.3(741.75 to 1670.91)	1.14(0.99 to 1.3)
Southern Latin America	55.64(36.06 to 77.2)	54.3(34.33 to 76.71)	-0.12(-0.28 to 0.01)	896.06(596.06 to 1235.89)	822.37(533.36 to 1146.08)	-0.35(-0.49 to -0.23)
Caribbean	36.68(23.45 to 52.21)	48.42(32.79 to 66.39)	0.97(0.87 to 1.07)	634.44(419.79 to 891.06)	834.89(573.01 to 1152.08)	0.97(0.88 to 1.06)
Central Europe	11.76(7.39 to 17.2)	12.69(8.16 to 18.14)	0.34(0.19 to 0.51)	236.21(163.63 to 324.06)	241.74(170.77 to 325.8)	0.06(-0.05 to 0.17)
Eastern Europe	1.66(0.97 to 2.53)	4.18(2.44 to 6.47)	2.89(2.36 to 3.26)	86.52(60.52 to 119.21)	120.91(83.98 to 168.14)	0.95(0.8 to 1.07)
Central Asia	1.14(0.57 to 2.03)	3.77(1.99 to 6.28)	3.99(3.75 to 4.24)	117.95(79.66 to 166.03)	164.34(112.25 to 227.58)	0.99(0.9 to 1.06)
North Africa and Middle East	77.15(46.67 to 129.33)	94.35(61.09 to 128.08)	0.72(0.67 to 0.76)	1254.08(774.07 to 2062.17)	1485.49(966.37 to 2019.84)	0.58(0.54 to 0.62)
South Asia	17.44(10.27 to 26.21)	21.22(12.76 to 31.8)	0.69(0.57 to 0.8)	375.33(247.81 to 531.01)	427.52(285.78 to 608.57)	0.49(0.43 to 0.57)
Southeast Asia	64.15(45.24 to 86.3)	84.16(59.76 to 110.45)	0.91(0.87 to 0.94)	1171.71(851.36 to 1551.9)	1476.9(1075.5 to 1915.09)	0.77(0.74 to 0.8)
East Asia	28.56(17.92 to 41.47)	24.75(15.48 to 36.31)	-0.41(-0.56 to -0.26)	528.45(360.66 to 734.08)	447.2(303.91 to 625.93)	-0.51(-0.57 to -0.44)
Oceania	13.4(7.17 to 22.65)	16.61(9.52 to 27.17)	0.64(0.59 to 0.69)	304.25(194.69 to 459.25)	360.73(237.97 to 539.52)	0.51(0.48 to 0.54)
Western Sub-Saharan Africa	96.82(67.35 to 128.53)	110.12(76.55 to 144.95)	0.43(0.4 to 0.46)	1569.58(1090.92 to 2078.74)	1723.42(1199.46 to 2264.47)	0.29(0.26 to 0.31)
Eastern Sub-Saharan Africa	62.87(37.76 to 94.18)	62.79(38.66 to 91.89)	0.02(-0.01 to 0.05)	1087.28(664.5 to 1613.55)	998.03(622.18 to 1459.5)	-0.26(-0.29 to -0.23)
Central Sub-Saharan Africa	97.45(61.1 to 142.96)	101.96(59.42 to 153.51)	0.13(0.11 to 0.16)	1643.02(1042.39 to 2402.78)	1673.5(1003.75 to 2504.56)	0.04(0.03 to 0.06)
Southern Sub-Saharan Africa	47.88(32.45 to 69.43)	97.57(69.7 to 129.25)	2.31(2.24 to 2.39)	794.17(554.69 to 1119.61)	1568.56(1131.64 to 2059.29)	2.21(2.13 to 2.27)

AAPC, the Average Annual Percentage Change (AAPC); ASDR, age-standardized disability-adjusted life year (DALYs) rates.

From 1990 to 2021, the global ASIR (AAPC = 0.63%, 95% CI: 0.62% to 0.63%, p<0.001), ASMR (AAPC = 0.99%, 95% CI: 0.94% to 1.02%, p<0.001), and ASDR (AAPC = 0.77%, 95% CI: 0.73% to 0.80%, p<0.001) showed upward trends, whereas the ASPR (AAPC = –0.25%, 95% CI: –0.26% to –0.24%, p<0.001) demonstrated a slight decline (**Tables 1**, **2**). Trends in disease burden and changes from 1990 to 2021 across SDI regions and the 21 GBD regions are also detailed in **Tables 1**, **2**. Notably, high SDI regions exhibited the highest ASPR (1857.79 per 100,000, 95%CI: 1654.77 to 2076.5) and ASIR (126.53 per 100,000, 95%CI: 95.62 to 162.01), while the highest ASMR (58.58 per 100,000, 95%CI: 38.57 to 80.55) and ASDR (972.72 per 100,000, 95%CI: 653.09 to 1333.43) were observed in low SDI regions in 2021.

**Figure 1:**
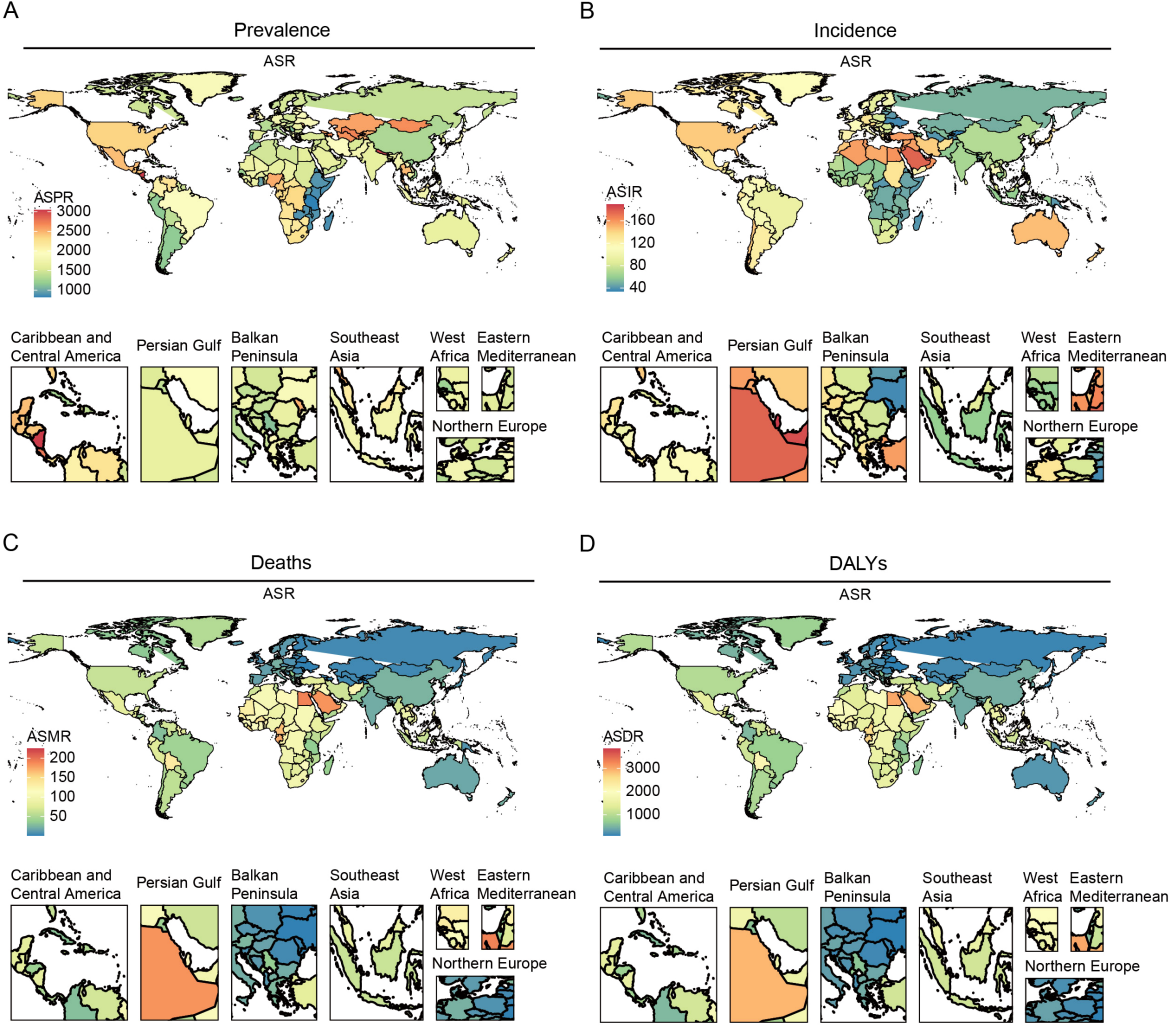
The global burden of the older HKD for both sexes in 204 countries and territories in 2021. **(A)** ASPR; **(B)** ASIR; **(C)** ASMR; **(D)** ASDR.

In 2021, the global ASPR of HKD varied widely, ranging from approximately 1,486.02 to 1,876.68 per 100,000 population. Nepal, Nicaragua, Costa Rica, and Mongolia recorded the highest ASPRs, each exceeding 2,700 per 100,000, whereas Eritrea, Madagascar, and Tanzania exhibited the lowest rates (<900 per 100,000) (**Figure 1A**, **Supplementary Table S2**).

Substantial changes in ASIR were observed between 1990 and 2021: according to Joinpoint regression analysis, Estonia, Hungary, and Armenia experienced the most rapid increases (e.g., Estonia AAPC = 2.93%, 95%CI: 2.88% to 2.98%), while Greece, Sweden, and Ireland showed slight decreases (**Figure 1B**, **Supplementary Tables S3, S4**). For ASMR, São Tomé and Príncipe, Mauritius, and Egypt had the highest mortality rates (>190 per 100,000), whereas Kyrgyzstan, Moldova, and Belarus had the lowest (<3 per 100,000) (**Figure 1C**, **Supplementary Table S5**).

Similarly, the ASDR was highest in Mauritius, São Tomé and Príncipe, and Egypt. Significant upward trends in ASDR were noted in Estonia and El Salvador, while noticeable declines were observed in the Maldives and Cyprus (**Figure 1D**, **Supplementary Tables S6, S7**). All estimates are age-standardized to the GBD global standard population and reported per 100,000 persons with corresponding 95% CIs. Detailed results for all 204 countries and regions are provided in the **Supplementary Tables**.

### Global disease burden of HKD by age and gender

**Figure 2:**
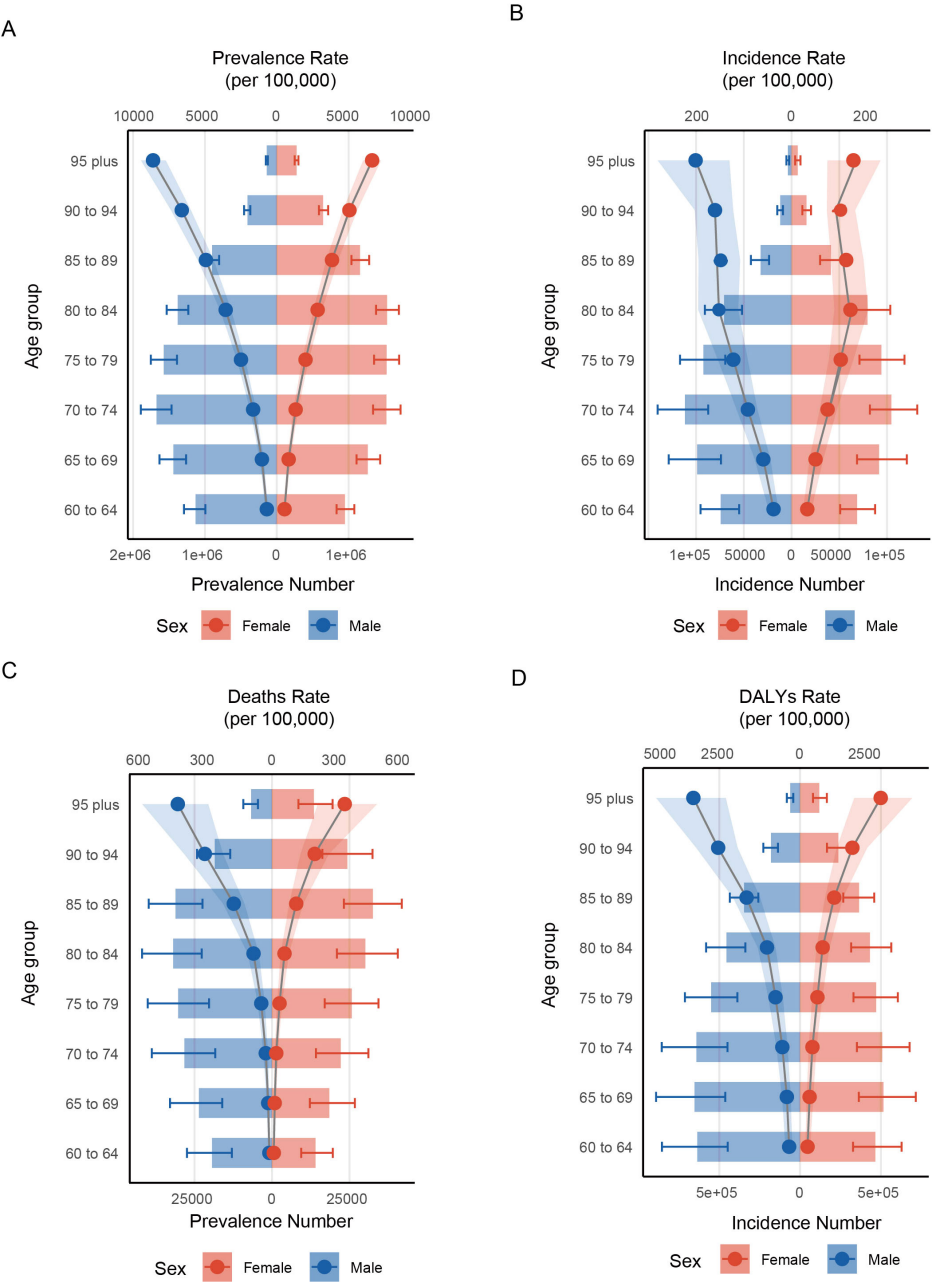
Distribution of rates across different age groups and between genders. **(A)** prevalence; **(B)** incidence; **(C)** deaths; **(D)** DALYs.

In 2021, global statistics showed that males in 60–79 had a higher prevalence number of HKD than their female counterparts (e.g., 60-64: male (1129378.44 cases, 95%UI: 992602.35 to 1291111.56) vs female (949837.43 cases, 95%UI: 833635.92 to 1081431.60)) In contrast, females exhibited greater prevalence in all other age brackets (**Figure 2A**, **Supplementary Table S8**). When examining incidence cases, males younger than 75 years consistently showed higher values than females, though this pattern reversed in older age groups (e.g., 70-74: male (111646.88 cases, 95% UI 87283.32 to 140326.66) vs female (104615.0386 cases, 95%UI: 82055.20652 to 131709.47); 75-79: male (92352.36 cases, 95%UI: 69409.25 to 116833.65) vs female (94124.69 cases, 95%UI: 71201.42 to 118320.68)) (**Figure 2B**, **Supplementary Table S9**). As for mortality and DALY metrics, males under 85 years of age bore a heavier burden than females (e.g., Mortality: 80-84: male (31905.16 cases, 95%UI: 22688.43 to 41922.19) vs female (30136.94 cases, 95%UI: 20946.38 to 40612.78); DALY: 80-84: male (455365.56 cases, 95%UI: 338575.69 to 581498.60) vs female (432877.80 cases, 95%UI: 315947.37 to 564539.24)) (**Figures 2C, D**, **Supplementary Tables S10, S11**). Overall, across the full age spectrum, males tended to have higher prevalence, incidence, mortality, and DALY rates (e.g. 95 plus: prevalence (males (9109.58 per 100,000 persons, 95%UI: 8173.02 to 10008.48) vs female (7031.48 per 100,000 persons, 95%UI: 6366.91 to 7696.84)); incidence (males (254.49 per 100,000 persons, 95%UI: 164.69 to 355.29) vs female (164.18 per 100,000 persons, 95%UI: 95.21 to 236.51)); mortality (males (444.58 per 100,000 persons, 95%UI: 300.54 to 613.11) vs female (343.29 per 100,000 persons, 95%UI: 216.24 to 496.25)); males (4005.92 per 100,000 persons, 95%UI: 2786.71 to 5404.78) vs female (3029.86 per 100,000 persons, 95%UI: 2033.11 to 4217.30)). Importantly, for both sexes aged 60 and above, all these indicators rose steadily with advancing age (e.g., for male in 60-64, the prevalence rate was 726.12 per 100,000 persons, 95%UI: 638.18 to 830.10) and the rate increased to 9109.5, 95%UI: 8173.02 to 10008.48 for those in 95 plus).

### Trends in the burden of HKD among older adults across different regions

**Figure 3:**
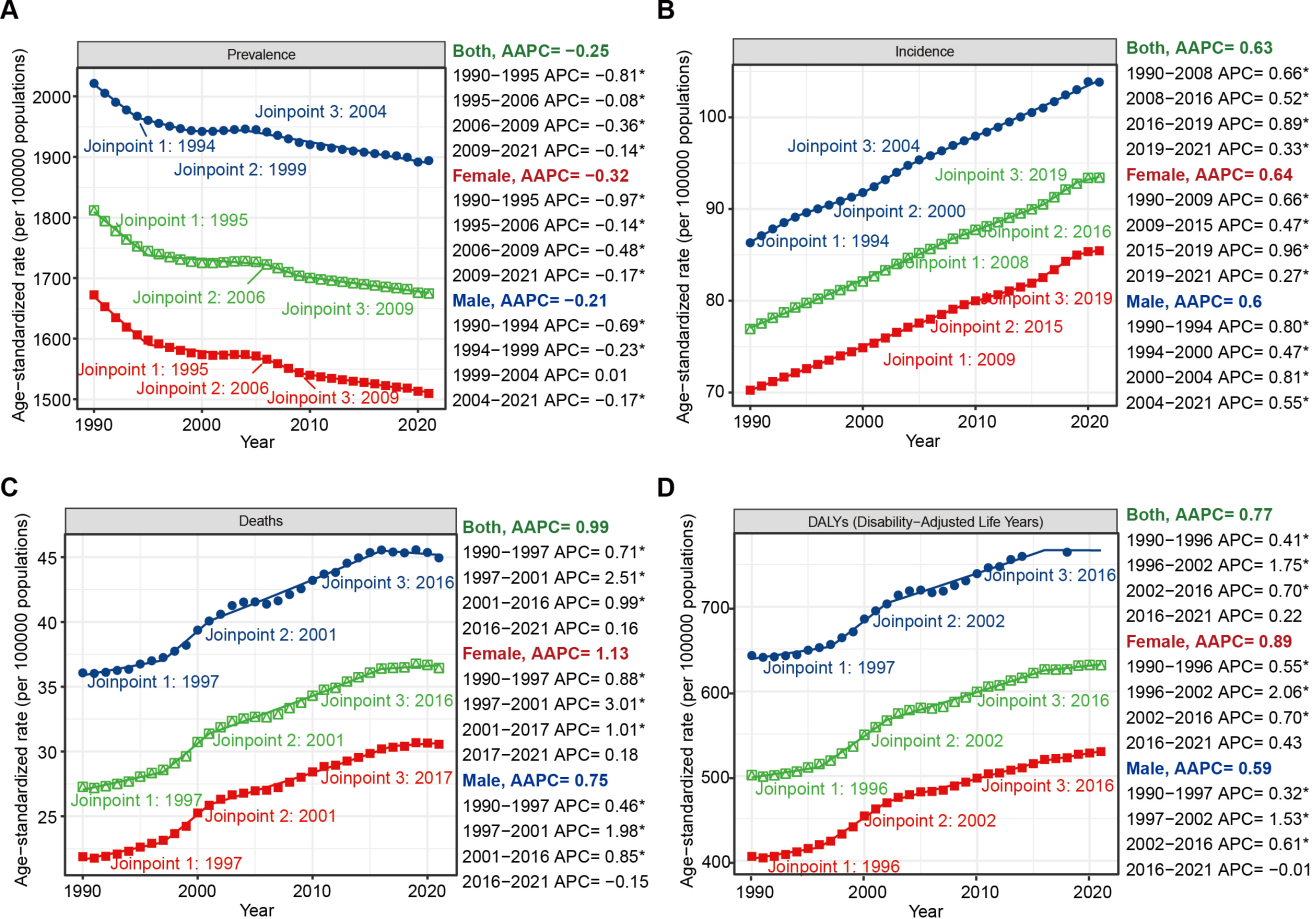
Joinpoint regression analysis of the temporal trends of HKD from 1990 to 2021: **(A)** ASPR; **(B)** ASIR; **(C)** ASMR; **(D)** ASDR. * means P-value < 0.05.

Global Joinpoint regression analysis revealed that over the past 32 years, the ASIR (AAPC = 0.63%; 95% CI: 0.62% to 0.63%; P < 0.001), ASMR (AAPC = 0.99%; 95% CI: 0.94% to 1.02%; P < 0.001), and ASDR (AAPC = 0.77%; 95% CI: 0.73% to 0.80%; P < 0.001) for HKD have shown overall upward trends, whereas the ASPR (AAPC = –0.25%; 95% CI: –0.26% to –0.24%; P < 0.001) has exhibited a downward trend. Similar trends were observed across different sex groups (**Figure 3**, **Tables 1**, **2**).

To evaluate the robustness of our trend segmentation, we performed a sensitivity analysis by increasing the maximum number of joinpoints from three to four in the global Joinpoint regression models. For incidence trends, the direction and statistical significance of AAPCs remained unchanged, and no additional joinpoints were detected, supporting the robustness of the model for this metric. However, for prevalence, mortality, and DALY trends, the sensitivity analysis yielded one additional joinpoint in each series and slight shifts in the timing of existing joinpoints (e.g., from 2005 to 2016 in the prevalence series). Despite these changes, the overall direction and statistical significance of trends across the entire study period remained consistent. These findings suggest that while precise breakpoints may vary slightly with model parameters, the general pattern and interpretation of long-term trends are robust. (**Supplementary Figure S1**, **Supplementary Tables S12, S13**).

At the SDI regional level, ASIR increased across all quantiles from 1990 to 2021 (e.g., AAPC = 0.37%, 95%CI: 0.34% to 0.39%, p<0.001, max joinpoint = 3). For ASMR and ASDR, an overall upward trend was observed, although a distinct transitional pattern occurred in low SDI regions, where significant declines were seen from 1995 to 2009, followed by marked increases from 2009 onward (ASMR: 1995-2009: APC = -0.70%, 95%CI: -0.80% to -0.62%, p<0.001; 2009-2015: APC = 1.67%, 95%CI: 1.38 to 2.21, p<0.001; ASDR: 1995-2009: APC = -0.78%, 95%CI: -0.84% to -0.73, p<0.001; 2009-2015: APC = 0.94%, 95%CI: 0.76% to 1.26%, p<0.001). Notably, while ASPR generally declined worldwide, high SDI regions experienced an accelerated decrease during 2019–2021 (APC = -0.86%; 95% CI: -1.19% to -0.43%) following a period of relative stability (**Supplementary Figures S2–S5**, **Supplementary Tables S14, S15**).

Age-stratified Joinpoint regression analyses also demonstrated that globally, prevalence rates were positively associated with age (**Supplementary Figure S5**). From 1990 to 2021, prevalence rates showed slight declines across all age groups, both globally and within different SDI regions (AAPC < 0, p < 0.001). In high SDI regions, the incidence rate among individuals aged 75–89 years was higher than that among those aged 90 years and older (**Supplementary Figure S6**). In contrast, mortality and DALY rates among individuals younger than 85 years remained relatively low (**Supplementary Figure S6**). For individuals aged 90 years and above, mortality and DALY rates exhibited a near-linear increase over the past 32 years, especially in those aged 95 years and older. Notably, during 2018–2021, a downward trend in mortality (APC = -4.81%, 95%CI: -6.03% to -2.88%, p<0.001) and DALYs (APC = -4.08%, 95%CI: -5.42% to -2.32%, p<0.001) was observed in those aged 95 years and above in high-middle SDI regions. In middle SDI regions, mortality and DALY rates remained stable throughout the 32-year period (**Supplementary Figures S5–S9**, **Supplementary Tables S16, S17**).

### Cross-country inequalities in HKD from 1990 to 2021

**Figure 4:**
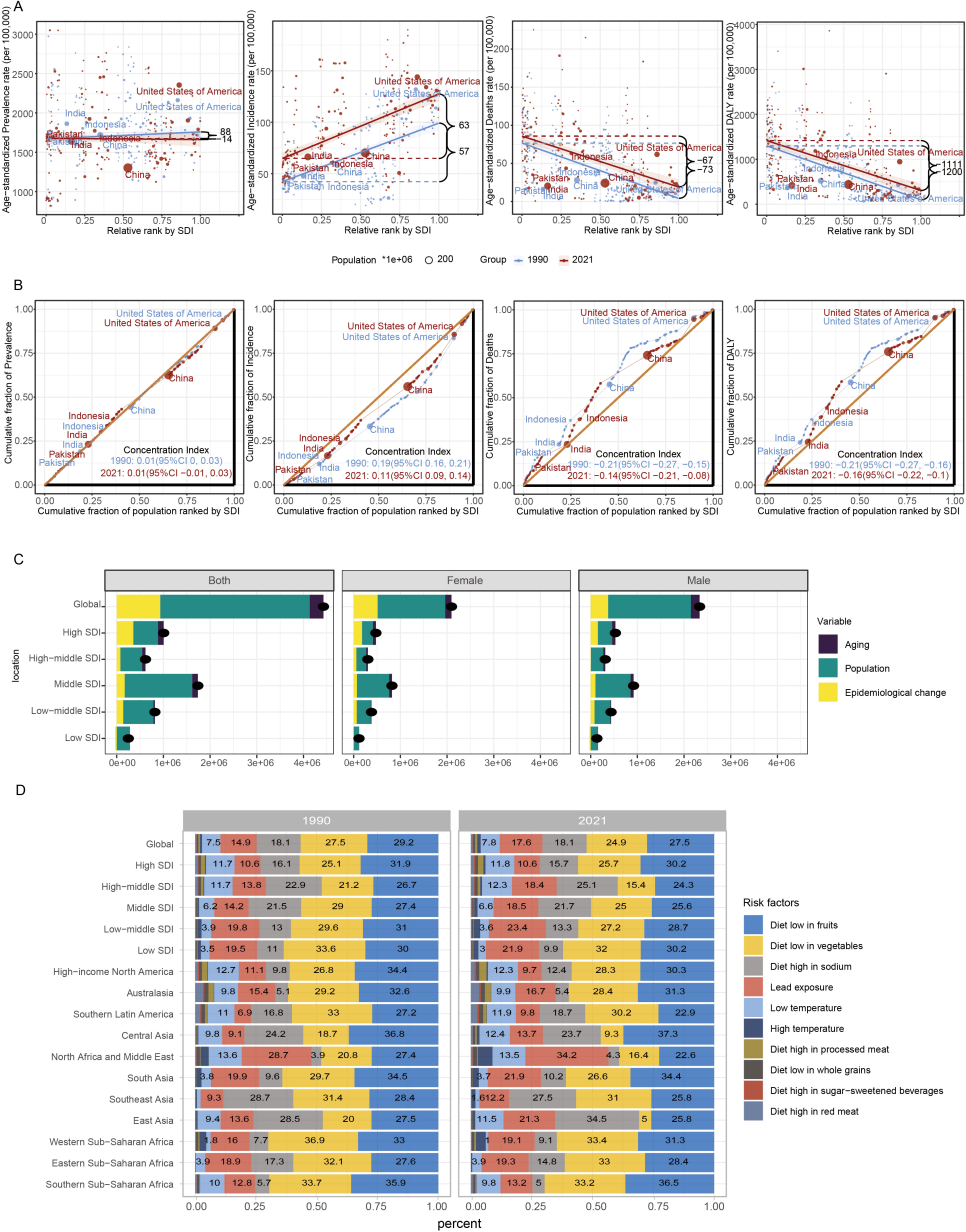
Global health inequality and risk factors of HKD in 1990 and 2021. **(A)** Health inequality regression curves for ASPR; ASIR; ASMR and ASDR. **(B)** Health inequality concentration curves for ASPR; ASIR; ASMR and ASDR. The top five most populous countries were highlighted; **(C)** Decomposition analysis of DALYs change in HKD from 1990 to 2021 at the global level and by SDI. **(D)** Contribution of diet and environment factors linked to HKD deaths in 1990 and 2021.

The burden of HKD exhibits significant absolute and relative inequalities across different levels of the SDI. From 1990 to 2021, the slope index for ASPR decreased from 88 (95%CI: -104 to 281) to –14 (95%CI: -184 to 156), indicating a narrowing disparity in prevalence among socioeconomic groups. However, the wide uncertainty intervals - encompassing both negative and positive values - indicate considerable statistical imprecision, and these results should be interpreted cautiously. In contrast, the slope index for ASIR increased from 57 (95%CI: 44 to 69) to 63 (95%CI: 51 to 74), suggesting a widening disparity in incidence. Regarding mortality and disability, the slope index for ASMR slightly improved, rising from –73 (95%CI: -88 to -58) to –67 (95%CI: -83 to -50), while the slope index for ASDR increased more notably from –1200 (95%CI: -1431 to -969) to –1111 (95%CI: -1372 to -850). These trends imply modest improvements in mortality disparities and substantial reductions in DALY disparities across socioeconomic groups, reflecting progress in addressing health inequalities (**Figures 4A–D**, **Supplementary Table S18**).

The concentration index for ASPR remained stable at 0.01 throughout the study period. However, the concentration index for ASIR notably decreased from 0.19 to 0.11, indicating a reduction in relative inequality in incidence. The concentration index for ASMR shifted from –0.21 (95% CI: -0.27 to -0.15) to –0.14 (95% CI: -0.21 to -0.08), and the concentration index for ASDR changed from -0.21 (95% CI: -0.27 to -0.16) to -0.16 (95% CI: -0.22 to -0.10), both reflecting decreasing relative inequalities in mortality and DALYs (**Figures 4E–H**).

In summary, while health inequalities related to HKD have decreased over the past few decades, improvements in disease management have been made. However, the relatively small changes in ASMR and ASDR highlight that further efforts are needed to continue reducing mortality and disability associated with HKD.

### Decomposition analysis of the burden of HKD

Decomposition analysis indicated a marked global increase in HKD-related DALYs from 1990 to 2021, with the most substantial growth occurring in regions with a middle SDI. The contributions to this rise were estimated at 6.66% from population aging, 72.31% from population expansion, and 21.03% from changes in disease patterns (**Supplementary Table S19**). Importantly, the relative impact of these factors differed among SDI categories, with population growth identified as the leading contributor to the escalating disease burden. These results highlight the central role of demographic expansion in driving the increased prevalence of HKD globally.

Further stratification by sex revealed comparable trends. In both male and female populations, population aging, growth, and epidemiological transitions were consistently associated with the rising number of DALYs, especially within middle SDI regions. This sex-specific analysis supports the overarching findings and underscores the broad demographic impact of these contributing factors (**Figure 4I**).

### Dietary and other risk factors for HKD-related deaths

This study outlines the proportion of HKD deaths attributable to specific risk factors from a global perspective, followed by regional trends from 1990 to 2021. The analysis primarily focuses on ASMR as the central metric to evaluate the global and regional burden of HKD. Globally, nearly 90% of HKD deaths in both 1990 and 2021 were attributable to a diet low in fruits, diet low in vegetables, diet high in sodium, and lead exposure. Among these, HKD deaths associated with a diet low in fruits remained the most dominant, with a death rate of 6.98 per 100,000 (95% CI: 3.66-10.74) in 2021. Deaths linked to a diet low in vegetables ranked second, with a death rate of 6.3 per 100,000 (95% UI: 3.22-10.13) (**Figure 4J**).

Distinct dietary risk profiles were observed across SDI regions: high, high-middle, and middle SDI regions were primarily affected by diets low in fruits, low in vegetables, and high in sodium, while lead exposure posed a significant risk in low-middle and low SDI regions.

Regionally, notable differences in HKD risk factor trends were observed. For instance, in North America and the Middle East, the proportion of HKD deaths attributed to lead exposure increased from 28.7% in 1990 to 34.2% in 2021, while deaths linked to a diet low in fruits and vegetables declined from 27.4% to 22.6% and from 20.8% to 16.4%, respectively. In East Asia, the proportion of HKD deaths due to a diet high in sodium rose from 28.5% to 34.5%, whereas those due to a diet low in vegetables decreased from 20% to 5% over the same period (**Figure 4J**).

### Frontier analysis of HKD

Frontier analysis identifies leading countries or territories whose SDI corresponds to the lowest burden of HKD. This minimal burden curve - referred to as the “efficiency frontier” - was constructed using non-parametric data envelopment analysis, with uncertainty captured through 1,000 bootstrap replications. The “effective difference” is defined as the distance from the frontier, representing the gap between a country’s observed burden and the minimal burden achievable at its SDI level (25). A significant effective difference indicates potential unrealized health benefits and opportunities for improvement based on a country’s or region’s position along the development spectrum.

**Figure 5:**
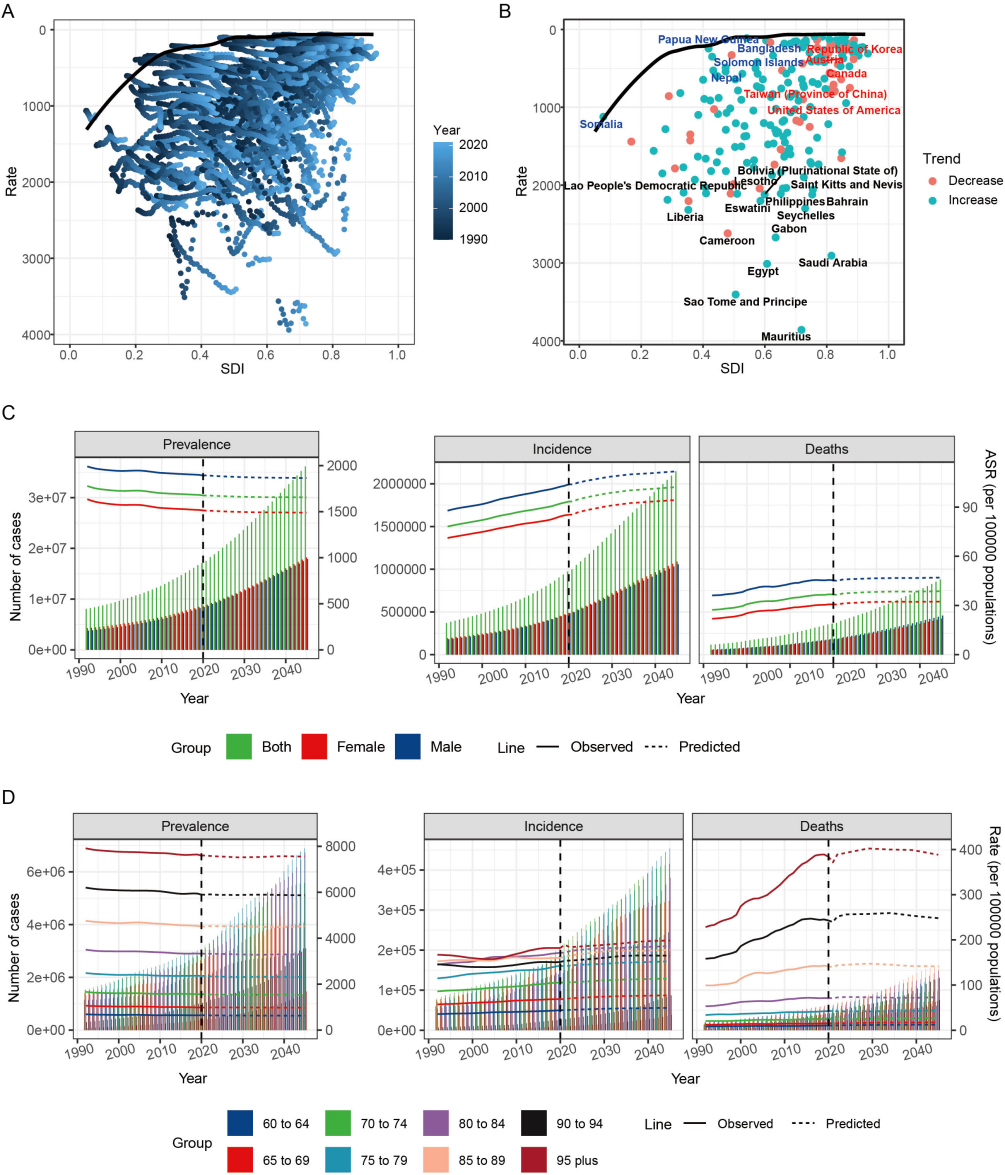
Frontier analysis and prediction of HKD burden by 2045. **(A)** Frontier analysis explores the relationship between ASDR and HKD from 1990 to 2021. **(B)** Frontier analysis of 2021. **(C)** Nordpred method predicted results of different age groups ASPR, ASIR and ASMR in HKD. **(D)** Nordpred method predicted results of different age groups of prevalence rate, incidence rate and mortality rate of HKD.

In countries with an SDI between 0.2 and 0.6, there was relatively large effective difference. However, as SDI increased beyond 0.6, these disparities tended to diminish (**Figure 5A**). From 1990 to 2021, ASDR trends showed an increase when SDI exceeded 0.8 (**Figure 5B**).

The top 10 countries or territories with the highest effective differences (243.09–350.95) were all located in middle and high-middle SDI regions, including Mauritius, Sao Tome and Principe, Egypt, Saudi Arabia, Gabon, Cameroon, Seychelles, Liberia, Eswatini, and Bahrain, suggesting substantial room for improvement. In contrast, the top 5 countries or regions with the lowest effective differences (all under 33) included Tajikistan (low-middle SDI), Ukraine (high-middle SDI), Finland (high SDI), Papua New Guinea (low SDI), and Belarus (high-middle SDI), indicating that they are at the forefront of reducing HKD burden (**Supplementary Table S20**).

Overall, the frontier analysis suggested that high SDI countries have relatively limited room for further improvement, while countries with lower SDI levels demonstrate greater potential for reducing the burden of HKD.

### Projected global burden of HKD by 2045

The global burden of HKD is projected to gradually increase. By 2045, it is estimated that there will be 36,138,049 prevalent cases of HKD, including 18,236,756 females and 17,901,294 males. Incidence cases are expected to reach 2,146,902, with 1,089,468 females and 1,057,434 males, while mortality is projected to be 879,295, with 421,847 females and 457,448 males (**Figure 5C**).

Changes in ASR for global epidemiological indicators by 2045 will vary. The ASPR is expected to be 1655.97, while the ASIR is projected to be 102.23, and the ASMR will be 38.67 per 100,000. Compared to 2021, the global ASPR (1674.93 per 100,000) is expected to decrease slightly by 2045, while both the ASIR (93.41 per 100,000) and ASMR (36.44 per 100,000) will experience slight increases (**Supplementary Table S21**). To assess the robustness and validity of the Nordpred projection model, we conducted a sensitivity analysis using a hold-out validation approach. Specifically, data from 1990 to 2016 were used to construct the prediction model, while the observed data from 2017 to 2021 were held out for comparison. The predicted values for 2021 closely approximated the actual observed rates: ASIR was predicted as 92 per 100,000 compared to the actual value of 93; ASDR was predicted as 37 per 100,000 versus the actual value of 36; and ASPR was predicted as 1,677 per 100,000 compared to the actual 2021 value of 1,674. These results support the stability and external validity of the Nordpred model in forecasting HKD burden (**Supplementary Figure S10**, **Supplementary Table S22**).

Age-stratified analyses indicate that the number of prevalent, incident, and death cases is expected to rise across all age groups by 2045, with the most significant increases among individuals aged over 90 years. The number of prevalent, incident, and death cases in this age group is projected to more than triple compared to 2021 levels. Conversely, for individuals under 75 years, the increase is expected to be less than two-fold. These findings highlight the particularly high disease burden among the elderly population and underscore the urgent need for strengthened management and preventive strategies targeting older adults (**Figure 5D**, **Supplementary Table S23**).

## Discussion

Hypertensive kidney disease poses a growing threat to global public health, particularly among aging populations (26). Leveraging GBD 2021 data, this study presents the first comprehensive analysis of the burden, drivers, and projected trends of HKD among individuals aged ≥60 years. Our findings underscore the increasing burden of HKD over the past three decades, particularly in low- and middle-SDI regions, and identify critical sociodemographic and modifiable risk factors underlying these trends.

The rise in ASIR alongside a modest decline in ASPR suggests improved detection and potentially better chronic disease management. The relatively high ASPR indicates a growing number of individuals living with HKD, likely reflecting expanded access to care, increased disease awareness, and improved survival. However, higher ASIR and ASPR in high-SDI regions may also reflect more aggressive screening policies, which can inflate disease detection compared to under-resourced settings where underdiagnosis persists (27, 28). Notably, evolving treatment strategies—such as the combination of RAAS blockers and SGLT2 inhibitors—have improved disease control in regions with access to these therapies, potentially modulating trends in prevalence and mortality (29, 30).

Marked regional differences further complicate the global landscape. While high-SDI regions benefit from declining mortality (ASMR) and disability (ASDR) trends - likely due to advances in hypertension management, dialysis, and pharmacotherapy - low-SDI regions show rising mortality and disability burdens. The observed decline in ASMR and ASDR between 1995 and 2009 in low-SDI areas, followed by reversal thereafter, may indicate a temporary benefit from international aid programs or epidemiologic transitions, with later resurgence driven by health system strain, demographic pressure, and poor control of noncommunicable diseases (NCDs) (31).

We identified survival bias in high-SDI countries, where individuals aged 75–89 had higher incidence than those ≥90 years. This could be due to healthier survivors or underdiagnosis in the oldest old, who often present atypical symptoms or receive limited diagnostic testing. These findings emphasize the importance of tailored screening and early intervention strategies, particularly targeting the “oldest young-old” (75–89 years), who are more likely to benefit from therapeutic intervention (32, 33). Gender disparities were evident, with males aged 60–74 exhibiting higher rates of HKD compared to females. Additionally, disease prevalence and mortality increased with age, peaking among individuals aged 85 and above. These patterns align with global trends identifying aging as a significant contributor to the rising burden of chronic conditions like HKD (34). Our Joinpoint sensitivity analysis further confirmed the robustness of trend detection: for incidence, no new joinpoints emerged when the maximum number was increased; for prevalence, mortality, and DALYs, small shifts (1–2 years) were observed in breakpoint timing, but the direction and significance of trends remained stable. These findings support the reliability of our reported AAPC trends and reduce concern about model-induced bias.

A major strength of our study lies in the integration of health inequality metrics. Although prevalence disparities narrowed between 1990 and 2021, incidence disparities widened (slope index: from 57 to 63), reflecting persisting inequities in early detection. Notably, while DALY-related disparities improved (slope index improved from -1200 to -1111), the 95% confidence intervals for prevalence-based slope indices were wide (e.g., from -104 to 281 in 1990), limiting our ability to draw definitive conclusions. Such wide uncertainty bounds likely reflect heterogeneous data quality and model sensitivity in underreported regions.

Our decomposition analysis attributes the rise in DALYs primarily to population growth (72.31%) and aging (6.66%), consistent with global demographic shifts. However, the impact of epidemiologic transition (21.03%) suggests that rising prevalence of risk factors (e.g., obesity, diabetes, poor diets) also plays a substantial role. Stratified by sex, these patterns persisted, affirming the generalizability of demographic drivers.

Our analysis highlights the significant influence of modifiable dietary and environmental factors on HKD-related mortality. In both 1990 and 2021, approximately 90% of HKD deaths globally were linked to preventable risk factors, including diets low in fruits and vegetables, excessive sodium intake, and lead exposure. Among these, insufficient fruit consumption remained the leading contributor to HKD mortality in 2021. The protective role of fruits and vegetables in kidney health is well-established. Higher intake of these foods is associated with a reduced risk of chronic kidney disease progression and lower all-cause mortality among individuals with kidney conditions. For instance, a Japanese hospital-based cohort study found that lower frequency of vegetable and fruit intake was significantly associated with higher risk of death, regardless of CKD status. These findings underscore the importance of dietary interventions in reducing the burden of HKD and improving patient outcomes (35). Similarly, a multinational cohort study involving hemodialysis patients demonstrated that increased consumption of fruits and vegetables was associated with a 20% lower risk of all-cause mortality (36). Conversely, excessive sodium intake is a known risk factor for hypertension and subsequent kidney damage. In East Asia, the proportion of HKD deaths attributed to high sodium diets increased from 28.5% in 1990 to 34.5% in 2021, underscoring the need for dietary sodium reduction initiatives. A systematic review of studies from August 2016 to March 2017 confirmed the negative effects of excessive sodium intake on health outcomes, including increased blood pressure and risk of kidney disease (37). Lead exposure has been identified as a significant environmental risk factor for HKD, especially in low and low-middle SDI regions. Chronic exposure to lead is associated with nephrotoxicity and increased mortality among individuals with kidney disease. A study in the New England Journal of Medicine found that environmental lead exposure accelerated the progression of chronic renal diseases in patients without diabetes (38). Furthermore, a global analysis based on the Global Burden of Disease Study 2019 reported that in 2019, there were approximately 52,940 deaths and 1.23 million DALYs of CKD caused by lead exposure, accounting for 3.71% of total CKD deaths (39). These findings emphasize the critical need for public health interventions focusing on dietary modifications and environmental health. Strategies such as promoting fruit and vegetable consumption, reducing sodium intake, and mitigating lead exposure through policy and infrastructure improvements are essential. Tailored interventions that consider regional dietary patterns and environmental exposures will be pivotal in reducing the global burden of HKD.

In our frontier analysis, countries with middle and high-middle SDI (e.g., Egypt, Mauritius) exhibited the largest “effective differences” (243–351), indicating the greatest potential for improvement given their development level. In contrast, countries like Tajikistan and Papua New Guinea had low effective differences, suggesting either lower disease burden or more efficient use of limited resources. The pattern suggests that economic advancement alone does not guarantee better outcomes-governance quality, health system efficiency, and prevention-oriented policies are crucial determinants.

Our projections indicate a modest increase in the ASIR and ASMR of HKD by 2045, particularly among older populations. This trend aligns with global patterns, suggesting that age-related conditions like HKD will continue to rise as the global population ages. Notably, individuals aged over 90 years are expected to experience the most significant increases in prevalence, incidence, and mortality, underscoring the urgent need for targeted management and preventive strategies for this demographic. Though Norpred modeling does not generate prediction intervals, we addressed this limitation by validating our model using historical back-casting, which showed good agreement between predicted and actual values for 2021. Our sensitivity analysis using historical (1990 - 2016) Norpred models to predict outcomes for 2017–2021 confirmed model robustness, with minimal deviation from observed values (e.g., ASIR: actual 93 vs predicted 92), increasing our confidence in forecast validity. Despite the challenges posed by the COVID-19 pandemic, the epidemiological trends for HKD remained largely unaffected, indicating the resilience of global health systems in managing chronic diseases during health crises. Advancements in medical technology and improved healthcare access are anticipated to mitigate some of the disease burden, particularly through enhancements in early diagnosis and treatment of kidney diseases. However, the projected increases in incidence and mortality rates highlight the necessity for continued efforts in HKD prevention and control, especially among the elderly. Future research should focus on developing effective prevention and management strategies to address this growing public health concern.

Several limitations warrant mention. First, the GBD estimation process is subject to uncertainty from data sparsity, especially in low-income countries. Although 95% UIs are reported for most indicators, decomposition and projection models lack formal uncertainty quantification. Second, while we present slope and concentration indices for health inequalities, some estimates have wide confidence intervals, limiting statistical precision. Third, our study uses an ecological approach - associating population-level indicators without individual-level data - which may obscure nuanced causal pathways. Finally, data harmonization challenges persist, particularly regarding age and diagnostic coding in regions with weak health information systems.

To mitigate these issues, future work should incorporate locally validated data sources (e.g., household surveys, cohort registries), develop Bayesian frameworks for uncertainty propagation in decomposition/projection models, and prioritize investment in surveillance infrastructure.

In conclusion, HKD represents an escalating global challenge, particularly among older adults. While incidence and mortality continue to rise, modifiable risk factors and persistent health inequalities offer actionable intervention points. Our findings highlight the urgency of prevention-oriented strategies - including dietary reform, environmental health investment, and equitable health system strengthening - to reduce the future burden of HKD.

## Data Availability

The original contributions presented in the study are included in the article/**Supplementary Material**. Further inquiries can be directed to the corresponding author.
