# Supplementary material for: Global burden, regional disparities, and future projections of hypertensive kidney disease in older adults: analysis of GBD 1990–2021 data
Source: Front Nephrol. 2025 Oct 17;5:1656865. doi: 10.3389/fneph.2025.1656865 (PMC12575384; doi:10.3389/fneph.2025.1656865)
Supplement: Supplementary file 1 [file DataSheet1.pdf]

Figure S1

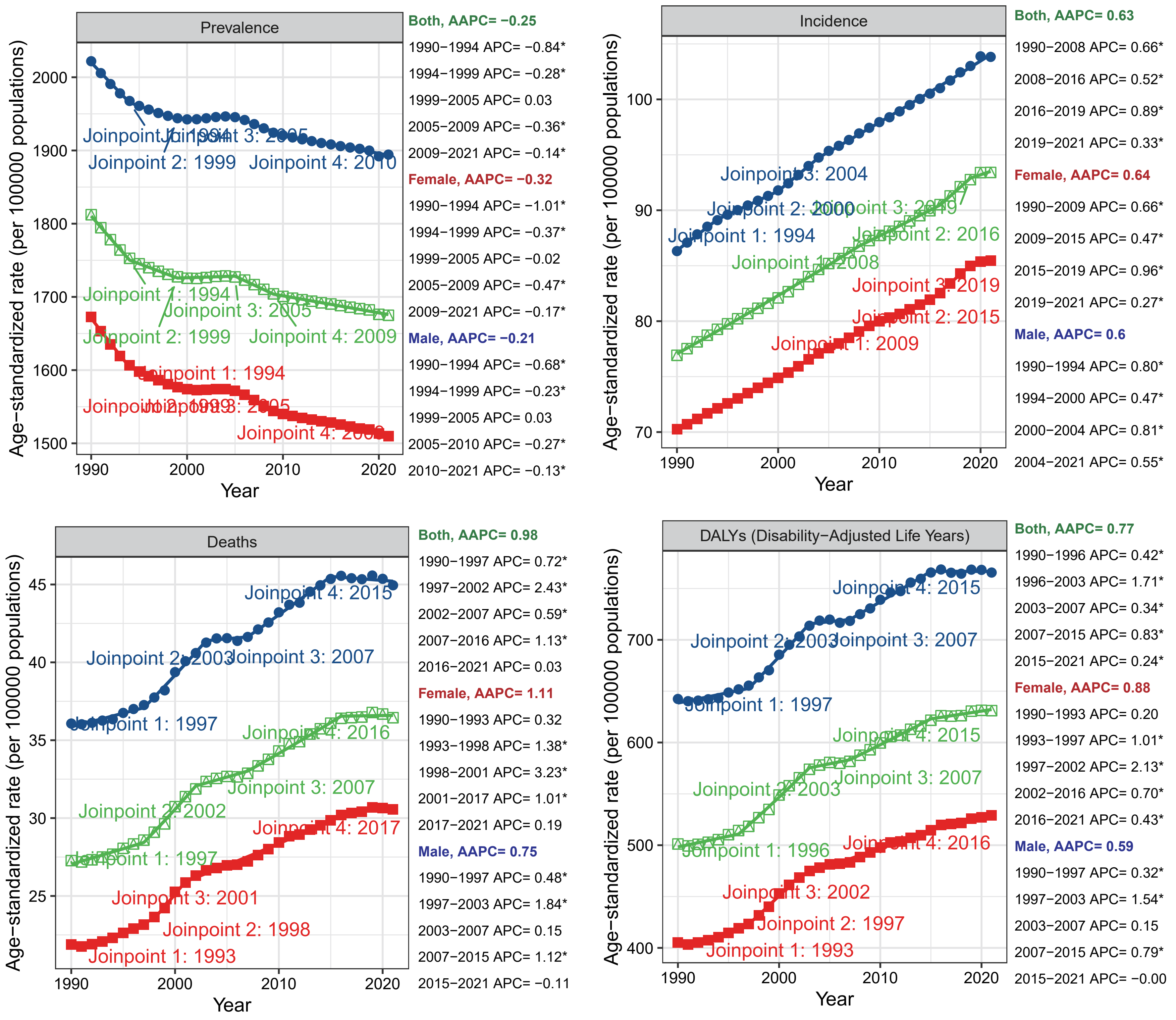

Figure S1 Sex-stratified joinpoint regression analysis of the temporal trends of HKD from 1990 to 2021 in global (max joinpoint =4)

Figure S2

High SDI

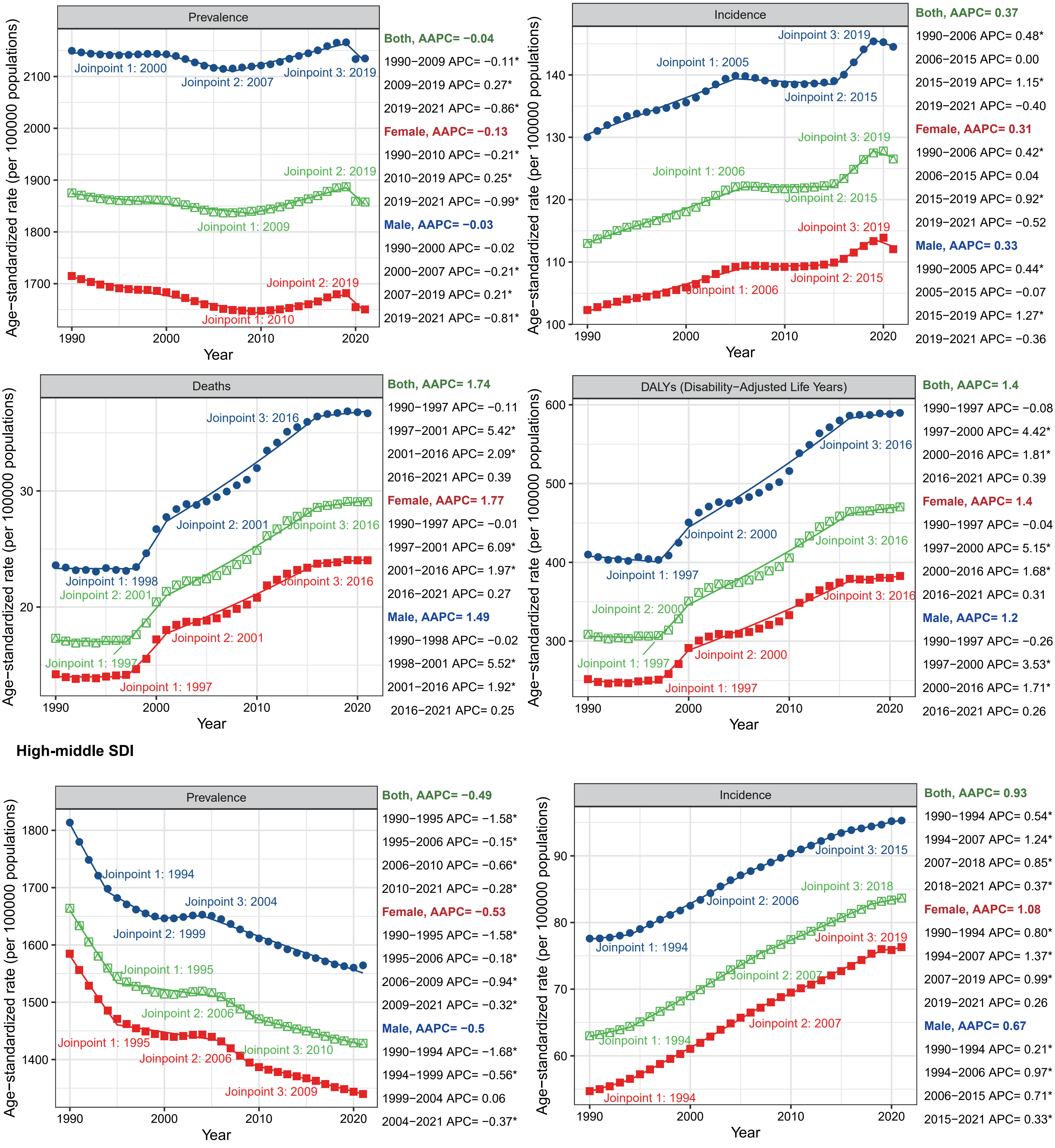

Figure S2 Sex-stratified joinpoint regression analysis of the temporal trends of HKD from 1990 to 2021 in high SDI and high-middle SDI regions.

Figure S3

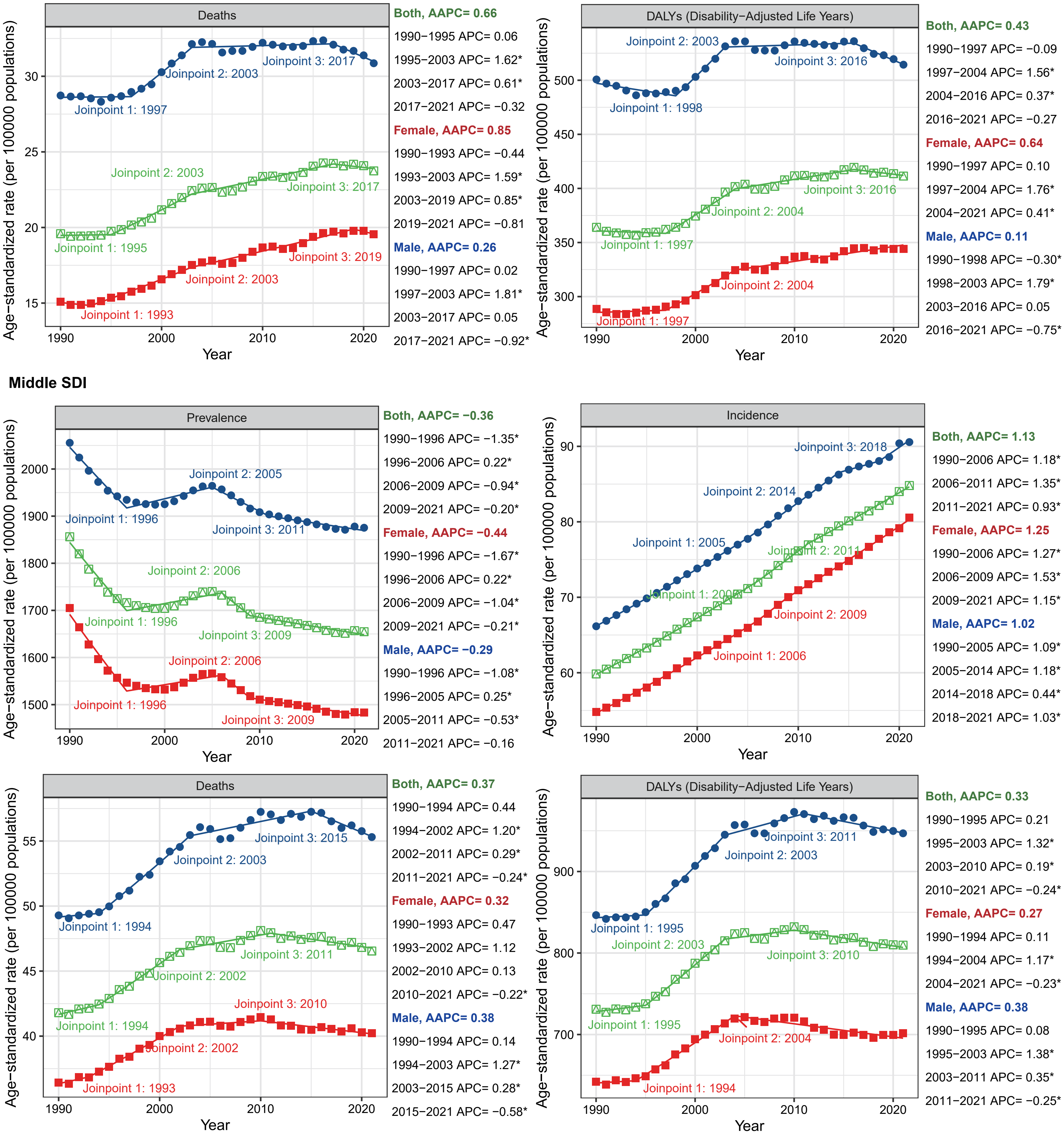

Figure S3 Sex-stratified joinpoint regression analysis of the temporal trends of HKD from 1990 to 2021 in high-middle SDI and middle SDI regions.

Figure S4

Low-middle SDI

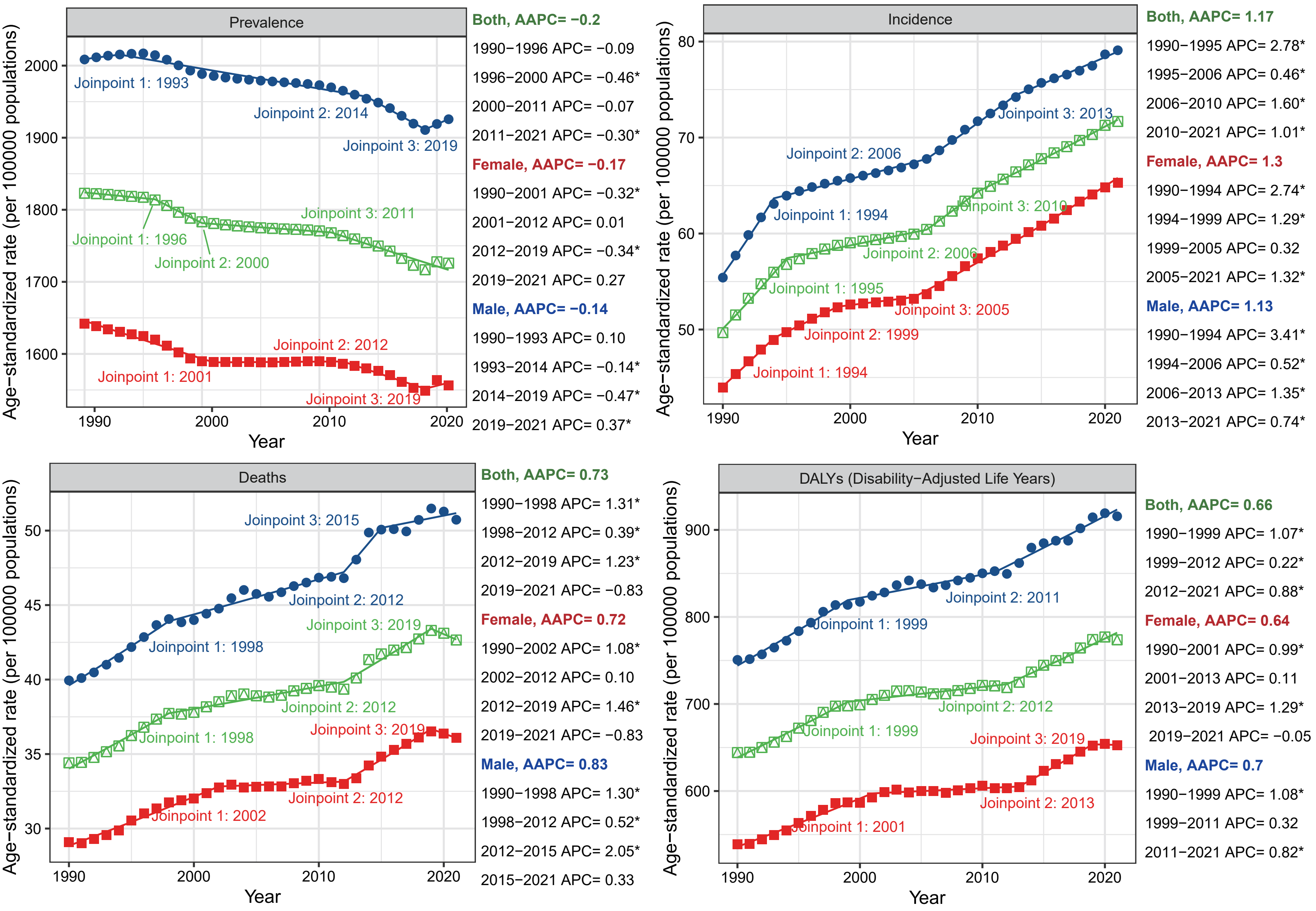

Low SDI

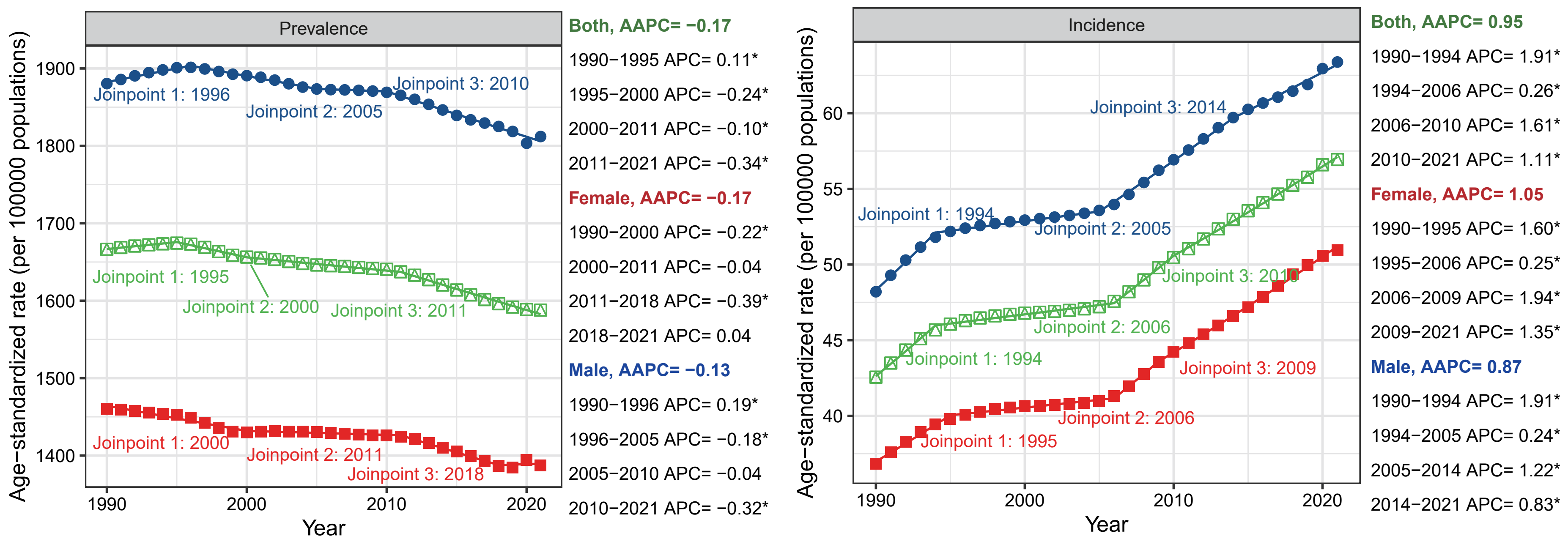

Figure S4 Sex-stratified **j**oinpoint regression analysis of the temporal trends of HKD from 1990 to 2021 in low-middle SDI and low SDI regions.

Figure S5

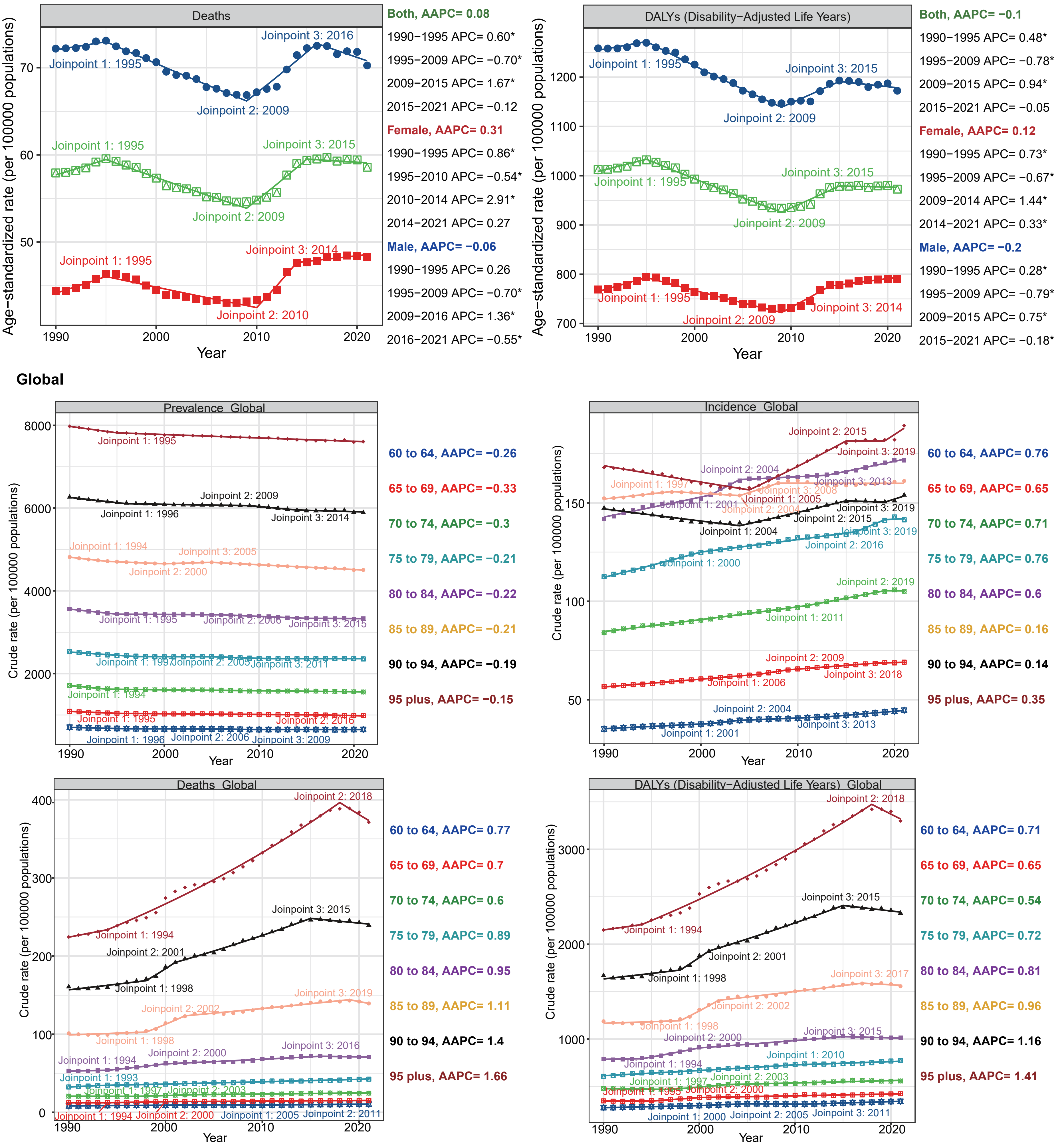

Figure S5 Sex-stratified joinpoint regression analysis of the temporal trends of HKD from 1990 to 2021 in low SDI regions and age-stratified joinpoint regression analysis in Global.

Figure S6

High SDI

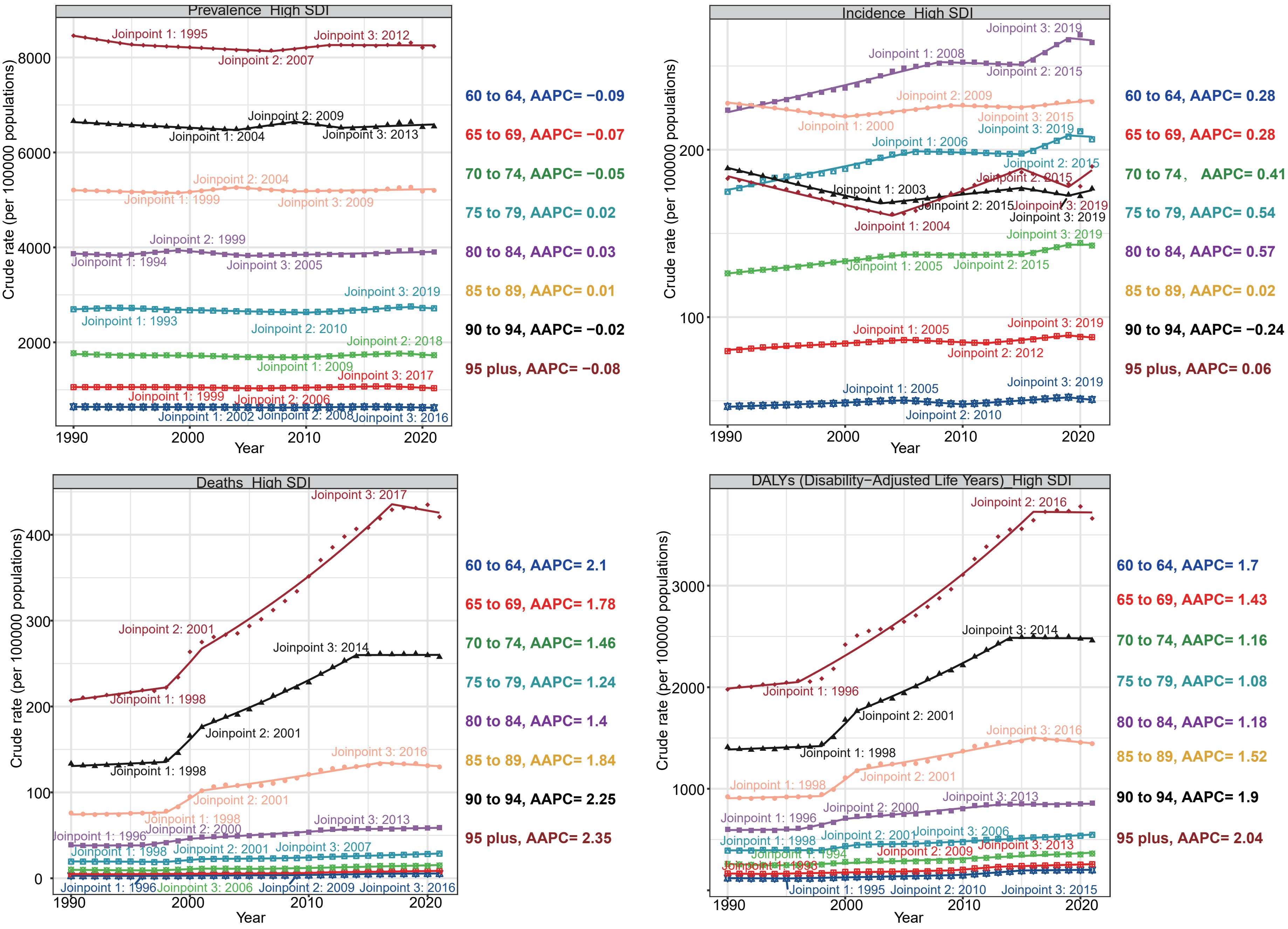

High-middle SDI

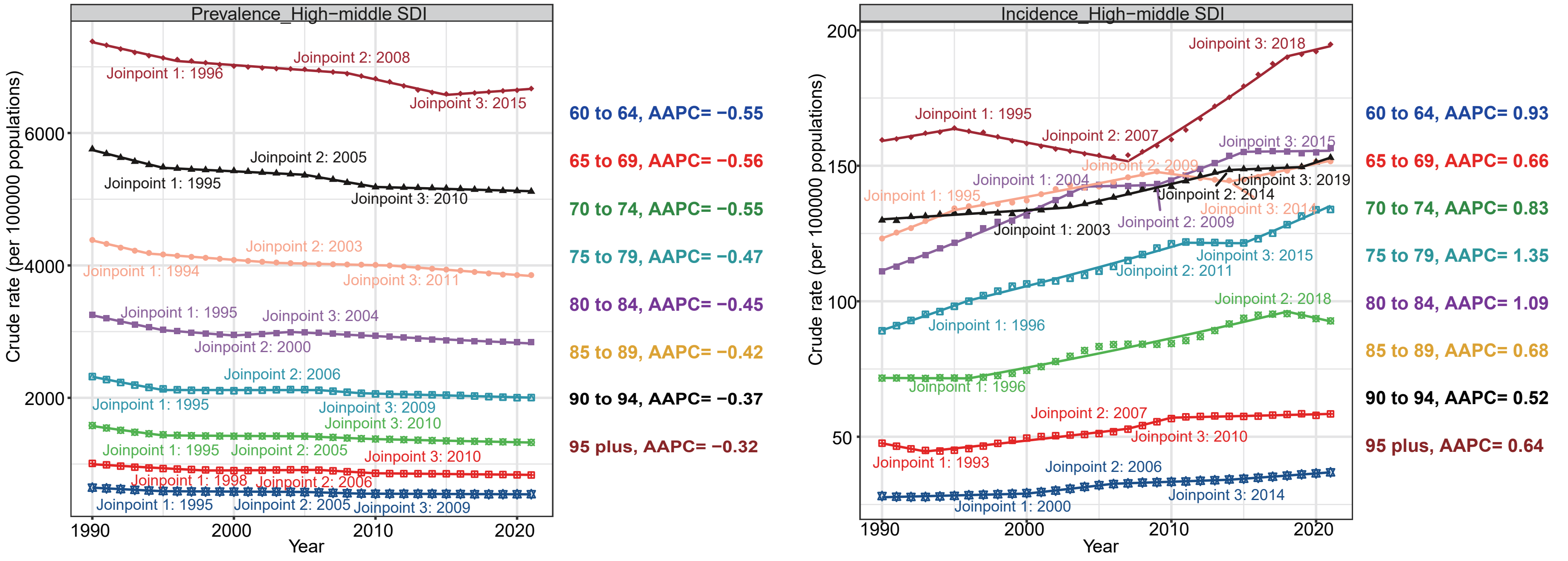

Figure S6 Age-stratified joinpoint regression analysis of the temporal trends of HKD from 1990 to 2021 in high SDI and high-middle SDI regions**.**

Figure S7

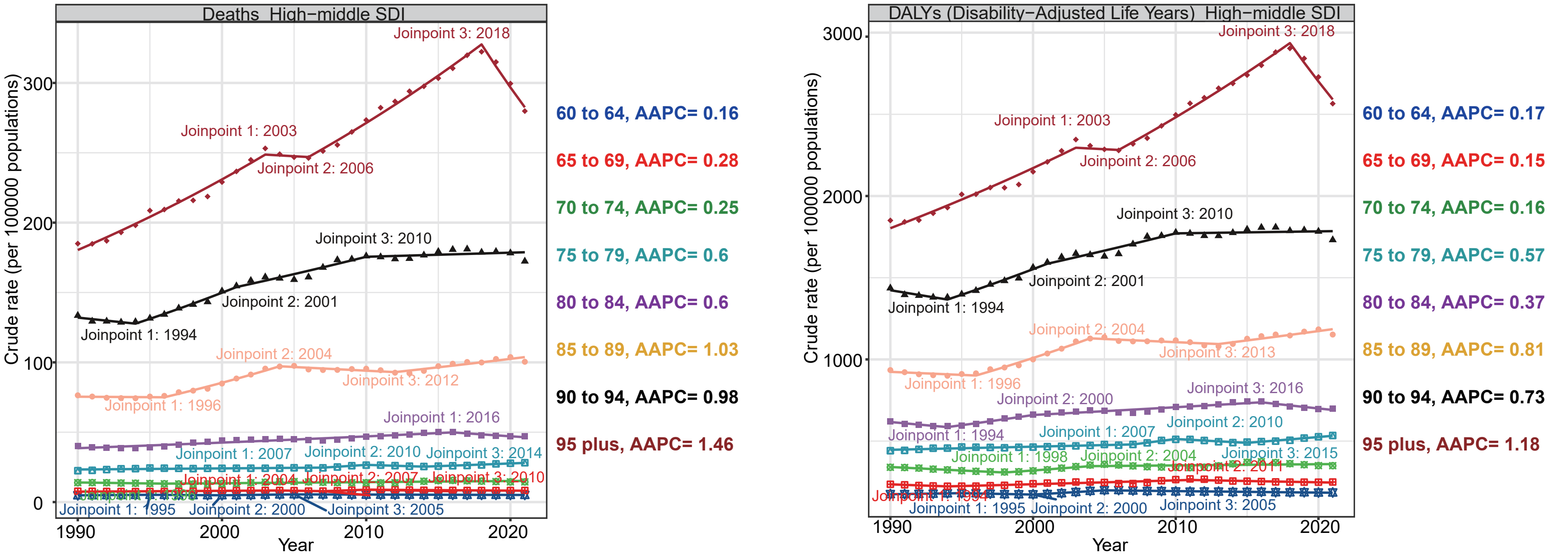

Middle SDI

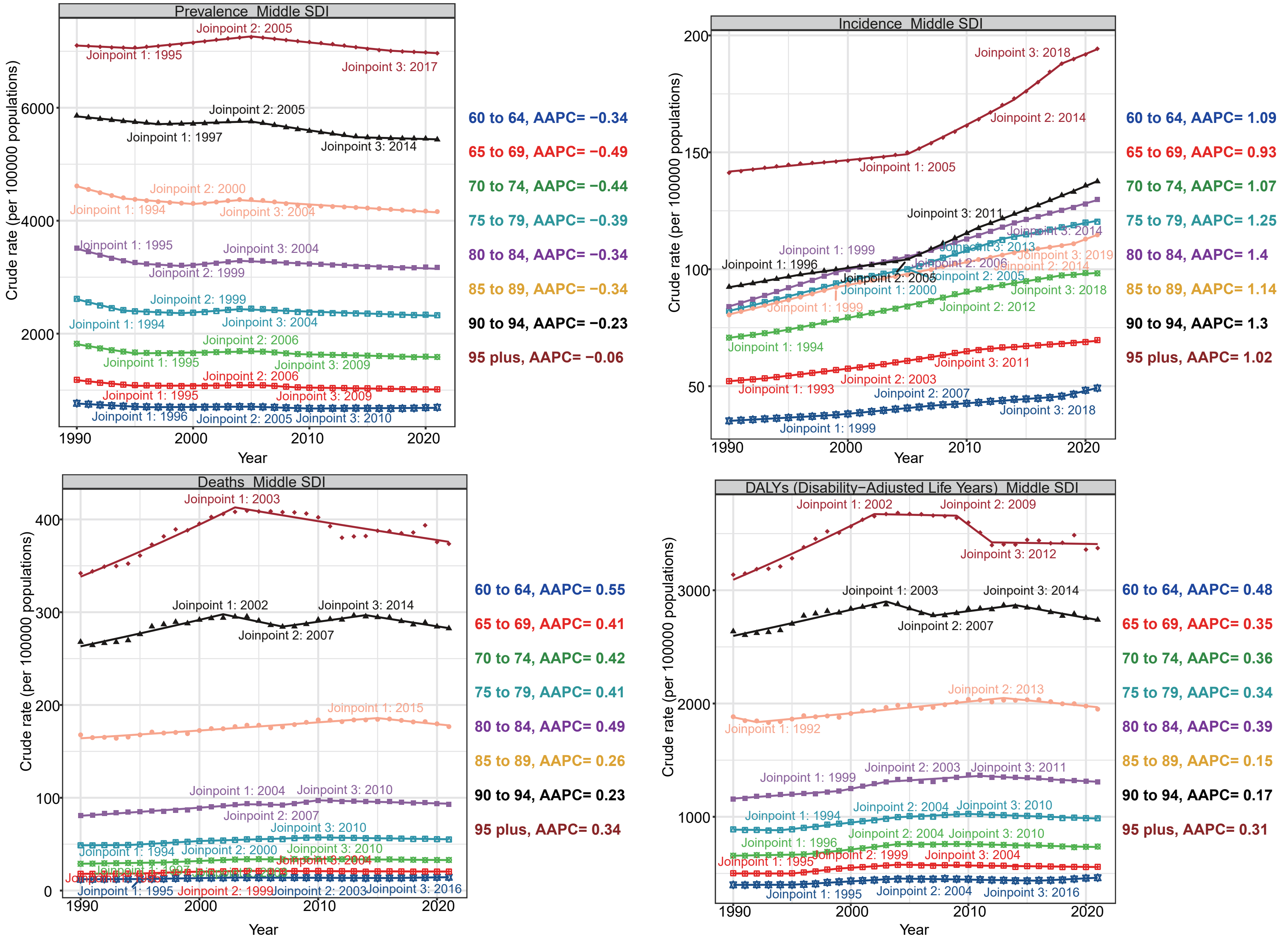

Figure S7 Age-stratified joinpoint regression analysis of the temporal trends of HKD from 1990 to 2021 in high-middle SDI and middle SDI regions.

Figure S8

Low-middle SDI

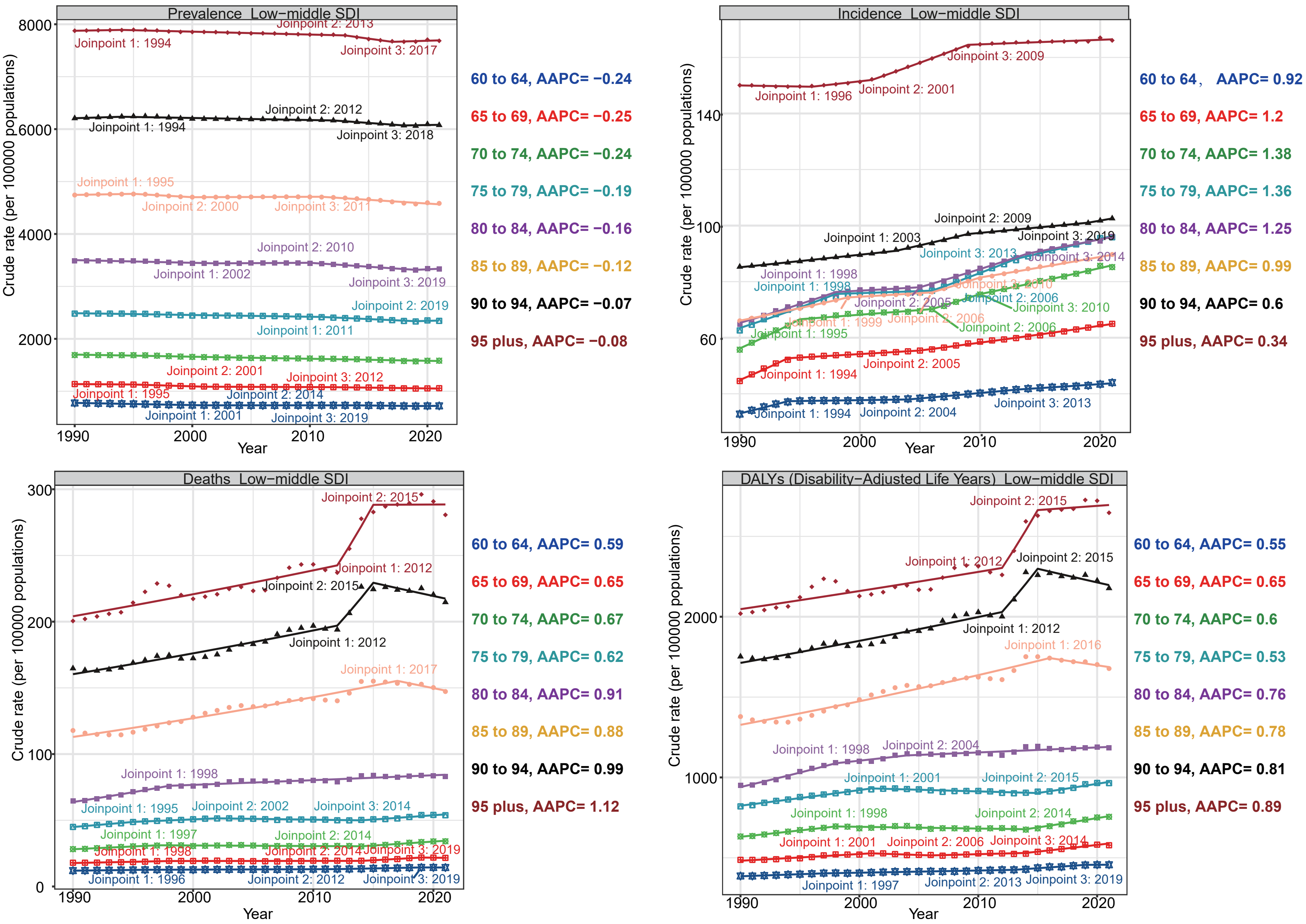

Low SDI

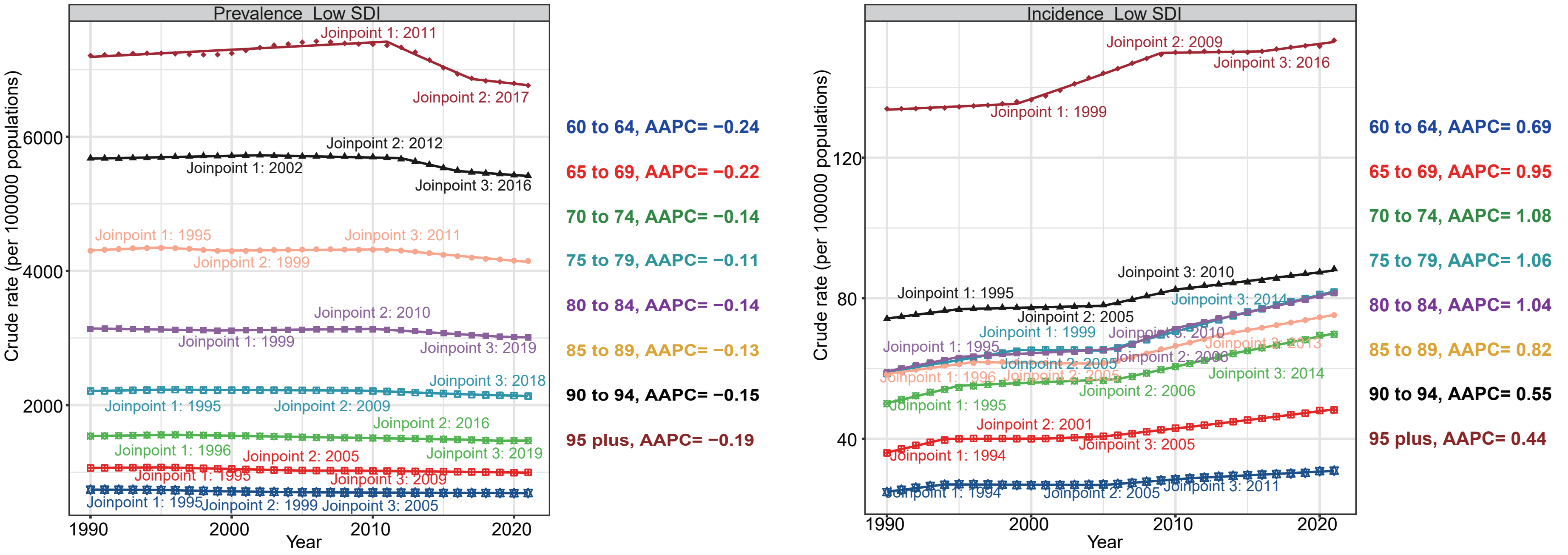

Figure S8 Age-stratified joinpoint regression analysis of the temporal trends of HKD from 1990 to 2021 in low-middle SDI and low SDI regions.

Figure S9

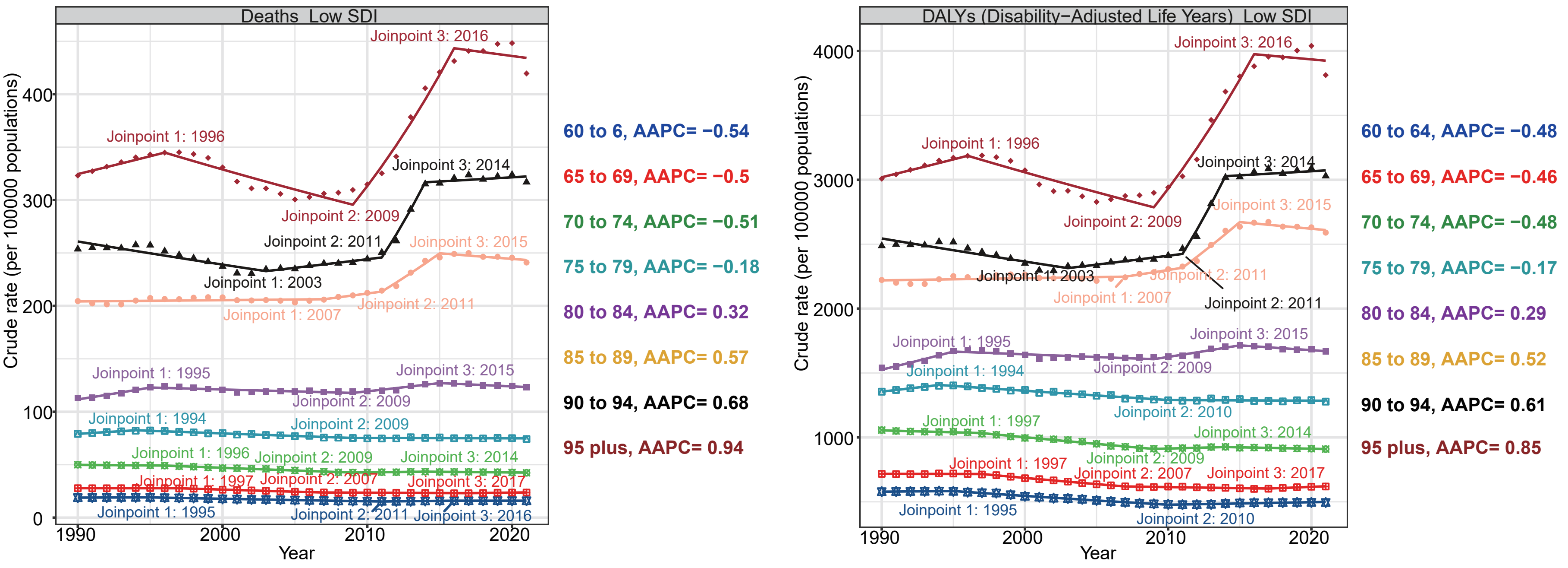

Figure S9 Age-stratified joinpoint regression analysis of the temporal trends of HKD from 1990 to 2021 in low SDI regions**.**

Figure S10

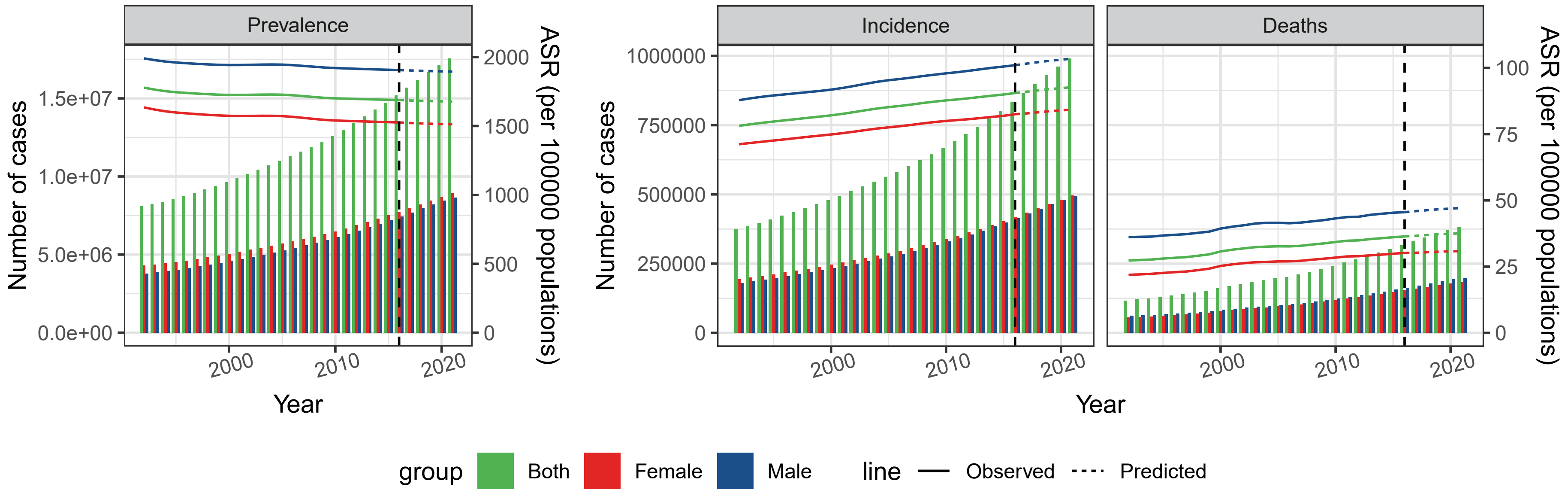

Figure S10 Nordpred method predicted results of different age groups ASPR, ASIR and ASMR in HKD.
